# *Mycobacterium tuberculosis* requires glyoxylate shunt and reverse methylcitrate cycle for lactate and pyruvate metabolism

**DOI:** 10.1111/mmi.14362

**Published:** 2019-08-23

**Authors:** Agnese Serafini, Lendl Tan, Stuart Horswell, Steven Howell, Daniel J. Greenwood, Deborah M. Hunt, Minh‐Duy Phan, Mark Schembri, Mercedes Monteleone, Christine R. Montague, Warwick Britton, Acely Garza‐Garcia, Ambrosius P. Snijders, Brian VanderVen, Maximiliano G. Gutierrez, Nicholas P. West, Luiz Pedro S. de Carvalho

**Affiliations:** ^1^ Mycobacterial Metabolism and Antibiotic Research Laboratory The Francis Crick Institute 1 Midland Road London NW1 1AT UK; ^2^ School of Chemistry and Molecular Biosciences The University of Queensland Brisbane 4072 Australia; ^3^ Bioinformatics and Biostatistics Science Technology Platform The Francis Crick Institute 1 Midland Road London NW1 1AT UK; ^4^ Mass Spectrometry Science Technology Platform The Francis Crick Institute 1 Midland Road London NW1 1AT UK; ^5^ Host‐Pathogen Interactions in Tuberculosis Laboratory The Francis Crick Institute 1 Midland Road London NW1 1AT UK; ^6^ Mycobacterial Research Program Centenary Institute of Cancer Medicine and Cell Biology Camperdown NSW 2050 Australia; ^7^ Department of Microbiology and Immunology, College of Veterinary Medicine Cornell University Ithaca NY USA

## Abstract

Bacterial nutrition is an essential aspect of host–pathogen interaction. For the intracellular pathogen *Mycobacterium tuberculosis* (Mtb), the causative agent of tuberculosis in humans, fatty acids derived from lipid droplets are considered the major carbon source. However, many other soluble nutrients are available inside host cells and may be used as alternative carbon sources. Lactate and pyruvate are abundant in human cells and fluids, particularly during inflammation. In this work, we study Mtb metabolism of lactate and pyruvate combining classic microbial physiology with a ‘multi‐omics’ approach consisting of transposon‐directed insertion site sequencing (TraDIS), RNA‐seq transcriptomics, proteomics and stable isotopic labelling coupled with mass spectrometry‐based metabolomics. We discovered that Mtb is well adapted to use both lactate and pyruvate and that their metabolism requires gluconeogenesis, valine metabolism, the Krebs cycle, the GABA shunt, the glyoxylate shunt and the methylcitrate cycle. The last two pathways are traditionally associated with fatty acid metabolism and, unexpectedly, we found that in Mtb the methylcitrate cycle operates in reverse, to allow optimal metabolism of lactate and pyruvate. Our findings reveal a novel function for the methylcitrate cycle as a direct route for the biosynthesis of propionyl‐CoA, the essential precursor for the biosynthesis of the odd‐chain fatty acids.

## Graphical abstract

*Mycobacterium tuberculosis* assimilates lactate and pyruvate utilising glyoxylate shunt and methylcitrate cycle, two pathways traditionally associated with fatty acid metabolism in bacteria. Surprisingly, we found that *M. tuberculosis* synthesises propionyl‐CoA by a reverse methylcitrate cycle and another partially characterised (in this study) pathway. Our results point to a re‐interpretation of the mechanistic role and utilisation of these pathways by Mtb during infection, i.e., their potential reversibility and importance during lactate and pyruvate metabolism *in vivo*.

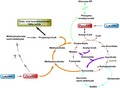

## Introduction

*Mycobacterium tuberculosis* (Mtb) is the causal agent of tuberculosis, a disease which causes ~1.5 million deaths every year. Tuberculosis is one of the top 10 leading causes of death worldwide (WHO, 2017). The discovery and understanding of the survival strategies employed by bacterial pathogens are essential to accelerate the development of novel and improved therapies. Bacterial nutrition is a key aspect of host–pathogen interaction (Olive and Sassetti, 2016; Ehrt *et al.*, 2018). Although drugs able to interfere with bacterial nutrition during infection might have profound effects on disease progression, no currently used antibiotics act by such a mechanism.

**Figure 1:**
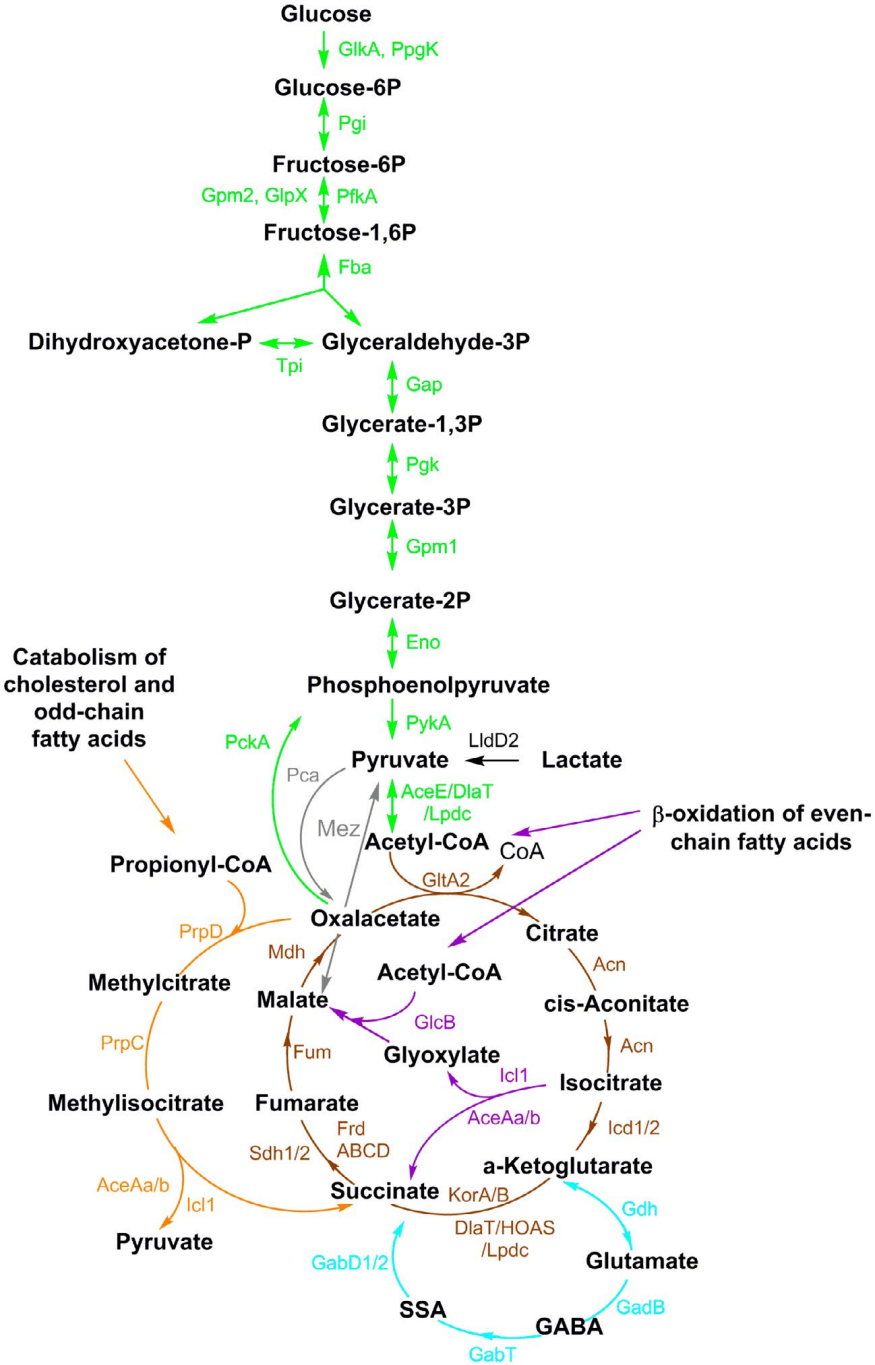
Part of the central carbon metabolism network (CCM) in Mtb. Scheme depicting enzymes and metabolites of some CCM pathways. Gluconeogenesis and glycolysis in green; Krebs cycle in brown; glyoxylate shunt in violet; methylcitrate pathway in orange; GABA shunt in light blue; anaplerotic (Mez) and cataplerotic reactions (Mez and Pca) in grey. The directionality of pathways is indicated on the base of current scientific knowledge of bacteria grown in aerobic conditions. The enzymes are described in agreement with https://mycobrowser.epfl.ch/ *or otherwise indicated*. AceE (Rv2241): pyruvate dehydrogenase E1 component; AceAa/b (Rv1915/Rv1916): isocitrate lyase; Acn (Rv1475c): aconitase; DlaT (Rv2215): dihydrolipoamide acyltransferase, E2 component of pyruvate dehydrogenase; Eno (Rv1023): enolase; Fba (Rv0363c): fructose‐biphosphate aldolase; FrdABCD (Rv1552‐Rv1555): fumarate reductase; Fum (Rv1098c): fumarase; GabD1/2 (Rv0324c/Rv1731): succinate‐semialdehyde dehydrogenase; GabT (Rv2589): 4‐aminobutyrate aminotransferase; GadB (Rv3432c): glutamate decarboxylase; Gap (Rv1436): glyceraldehyde 3‐phosphate dehydrogenase; Gdh (Rv2476c): glutamate dehydrogenase; GlcB (Rv1837c): malate synthase; GlkA (Rv0650): glucokinase; GlpX (Rv1099c): fructose 1,6‐biphosphatase; GltA2 (Rv0896): citrate synthase; Gpm1 (Rv0489): phosphoglycerate mutase; Gpm2 (Rv3214): fructose 1,6‐biphosphatase; KorA/B (Rv2454c/Rv2455c)^**^: anaerobic α‐ketoglutarate dehydrogenase; HOAS (Rv1248c)^***^: 2‐hydroxy‐3‐oxoadipate synthase; Icd1/2 (Rv3339c/Rv0066c): isocitrate dehydrogenase; Icl1 (Rv0467): Isocitrate lyase; LldD2 (Rv1872c): lactate dehydrogenase; Lpdc (Rv0462): dihydrolipoamide dehydrogenase; Mez (Rv2332): malic enzyme; Mdh (Rv1240): malate dehydrogenase; Pca (Rv2967c): pyruvate carboxylase; PckA (Rv0211): phosphofructokinase; PfkA (Rv3010C): phospho‐fructokinase; Pgi (Rv0946): phospho‐glucose isomerase; Pgk (Rv1437): phosphoglycerate kinase; PpgK (Rv2702): polyphosphate glucokinase; PrpC (Rv1131): methyl‐citrate synthase; PrpD (Rv1130): methyl‐citrate dehydratase; PykA (Rv1617); pyruvate kinase; Sdh1/2 (Rv2047c‐Rv2049c/Rv3316‐3319): succinate dehydrogenase; Tpi (Rv1438): triosephosphate isomerase. GABA: 4‐aminobutyric acid. SSA: succinic semialdehyde. */**/***: see supplementary information.

Mtb is an intracellular pathogen that primarily infects macrophages, but also infects neutrophils (Eum *et al.*, 2010), lymphatic endothelial cells (Lerner *et al.*, 2016) and other cell types. It is commonly accepted that Mtb is adapted to use host lipids (accumulated in functional lipid bodies) during intracellular replication (Lovewell *et al.*, 2016). Host‐derived fatty acids can be incorporated into mycobacterial lipid inclusions, or directly incorporated in mycobacterial membranes (Lovewell *et al.*, 2016). Moreover, the ability of Mtb to catabolise cholesterol or fatty acids as carbon and energy sources *in vitro* suggests that lipids may be required during infection. In mycobacteria, the two final β‐oxidation products, acetyl‐CoA and propionyl‐CoA (derived only from odd‐chain fatty acids), are directed into the glyoxylate shunt and the methylcitrate cycle respectively. The first reaction of the glyoxylate shunt and the last reaction of the methylcitrate cycle are both catalysed by the *icl*‐encoded isocitrate lyase (Gould *et al.*, 2006) (Fig. 1). When present as sole carbon source, lipid‐derived carbon units are channelled towards gluconeogenesis by the *pckA*‐encoded phosphoenolpyruvate carboxykinase (Marrero *et al.*, 2010) (Fig. 1). The inability of *icl* and *pckA* knockout mutants to establish an infection in mice (Munoz‐Elias and McKinney, 2005; Marrero *et al.*, 2010) led to the hypothesis that lipids are the major carbon source for Mtb *in vivo*.

Although lipids are certainly used as carbon sources during infection, their status as major carbon source is somehow counterintuitive, given their very limited solubility and the abundance of other carbon sources. Mtb has been shown to co‐catabolise different carbon sources *in vitro* (de Carvalho *et al.*, 2010) and in macrophages (Zimmermann *et al.*, 2017). Also, the essentiality of isocitrate lyase in the absence of fatty acids suggests that this enzyme has important roles beyond fatty acid metabolism (Eoh and Rhee, 2013). Together, these observations suggest that there might be other important carbon sources utilised by Mtb during infection that remain to be identified.

Host‐derived terminal glycolytic products such as pyruvate and L‐lactate (lactate) have been under investigated in the context of Mtb infection. Pyruvate and lactate are commonly found reaching μM and mM concentrations in blood, muscle and epithelial mucosa as well as in cerebrospinal fluid (Zhang and Natowicz, 2013; Gu *et al.*, 2014; Mason *et al.*, 2016). Importantly, production and secretion of lactate increases during inflammation as a consequence of the switch from oxidative to fermentative metabolism (Kelly and O'Neill, 2015) and this phenomenon clearly occurs during macrophage infection by Mtb (Somashekar *et al.*, 2011; Shi *et al.*, 2015). Many pathogens can use lactate as a carbon source *in vitro*. It is well known that *Neisseria meningitidis* relies on host‐derived lactate to be virulent (Exley *et al.*, 2005; 2007; Sigurlasdottir *et al.*, 2017) and recently, it has been reported that host‐derived lactate also supports *Salmonella enterica* (Gillis *et al.*, 2018) virulence. Mtb can use lactate or pyruvate as sole carbon source *in vitro* (Youmans and Youmans, 1953) and Mtb LldD2/Rv1872c lactate dehydrogenase is essential for replication in human‐derived macrophages (Billig *et al.*, 2017). Although these studies suggest that lactate and pyruvate might represent important carbon sources for Mtb during infection, the metabolic pathways involved in their assimilation are only partially defined.

In this work, we identified the central metabolic pathways for lactate and pyruvate metabolism in Mtb by combining microbial physiology analysis with transposon‐directed insertion site sequencing (TraDIS) (Langridge *et al.*, 2009), RNA‐seq transcriptomics, proteomics and stable isotopic labelling coupled with mass spectrometry‐based metabolomics. Our results reveal the structure of Mtb's metabolic network associated with catabolism of pyruvate and lactate and their potential as host‐derived carbon sources.

## Results

### Lactate and pyruvate are superior carbon sources compared to glucose and fatty acids

**Figure 2:**
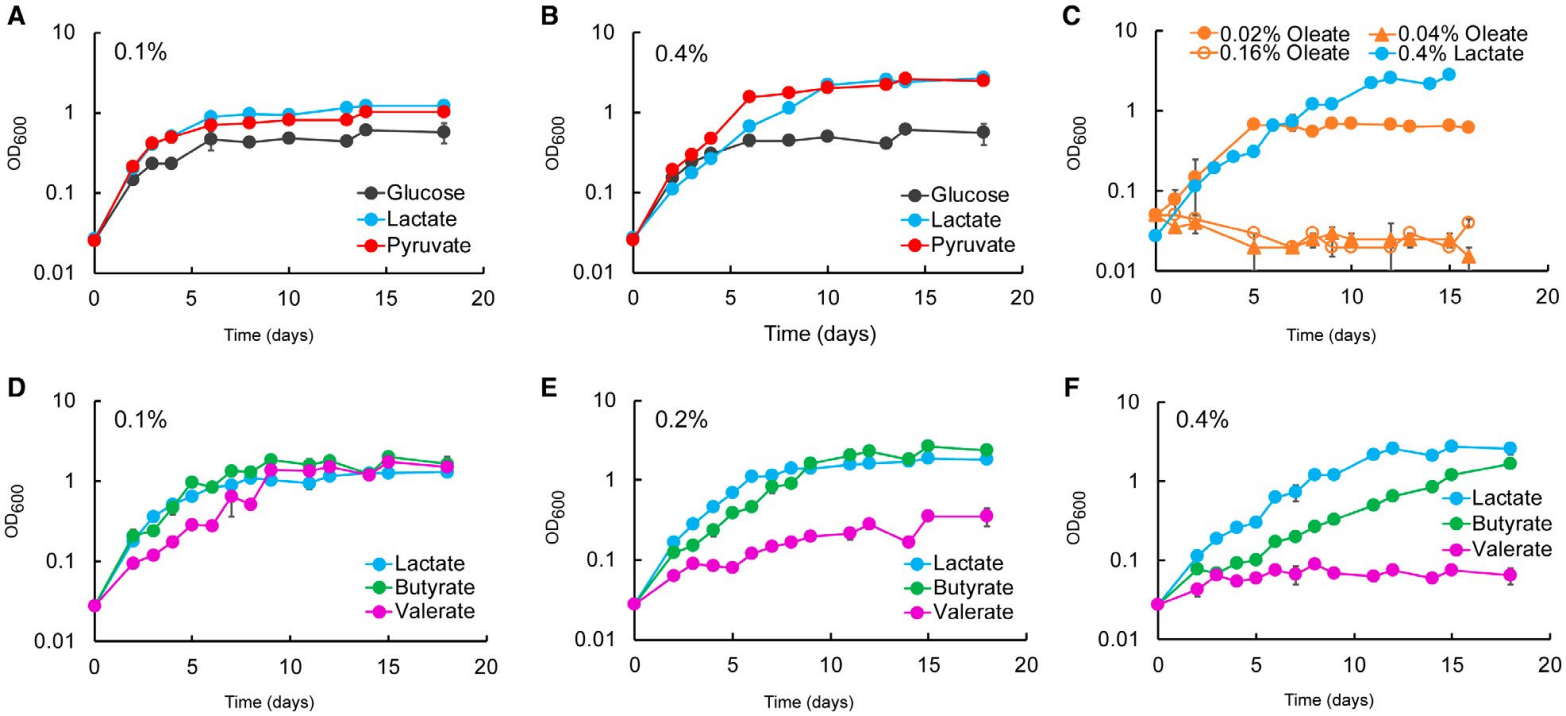
Lactate and pyruvate are superior carbon sources compared to glucose and fatty acids for Mtb. Growth of Mtb at different concentration of lactate, pyruvate, glucose (A and B) and fatty acids (C–F) as principal carbon sources. 0.4% of glucose (22.2 mM), lactate (44.4 mM) and pyruvate (45.2 mM) produce a similar number of acetyl‐CoA units. 0.16% oleate (5 mM) produces a similar number of acetyl‐CoA units of 0.4% lactate and pyruvate. The number of carbons increases from butyrate (C_4_), valerate (C_5_) to oleate (C_18_). The charts show the average and standard deviation from three independent experiments.

First, we investigated Mtb growth in lactate or pyruvate as principal carbon source, in comparison with glucose and fatty acids (Fig. 2). All cultures were pre‐adapted in matched carbon sources to prevent complications derived from ‘memory’ of the previous rich medium (de Carvalho *et al.*, 2010). Mtb replicates with similar rate in lactate and pyruvate with culture densities increasing proportionately with their supply (0.1–0.4%). On the contrary, Mtb replication in glucose is less efficient and does not improve from 0.1 to 0.4% carbon source (Fig. 2A and B), and the ability of Mtb to grow in fatty acids decreased with both increasing concentration and carbon chain length (Fig. 2C–F). These results indicate that Mtb can indeed utilise lactate or pyruvate as principal carbon sources more efficiently than glucose or fatty acids.

**Figure 3:**
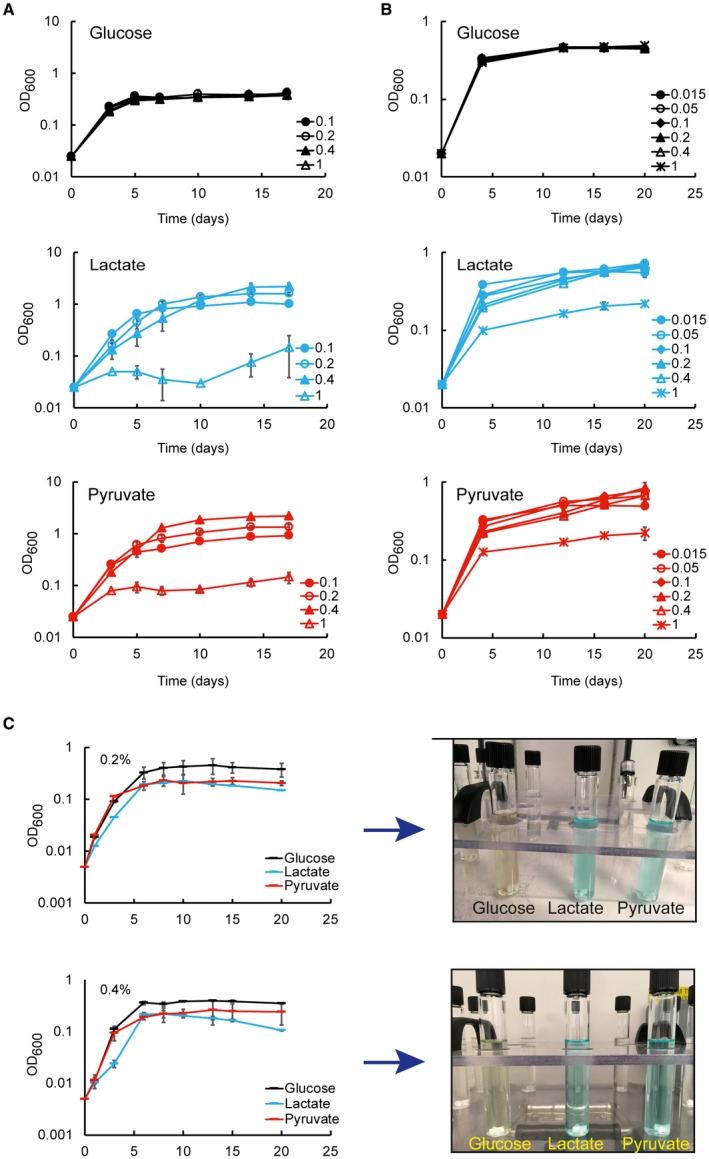
Limited aeration reduces the growth of Mtb in lactate and pyruvate. A. Growth of rolling cultures (30 rpm) with liquid/air volume ratio of about 1:5. The plots show the average and error (average deviation) of two independent experiments. B. Growth of standing cultures with liquid/air volume ratio of about 1:10. The plots show the average and errors (average deviation) of two independent experiments. C. Growth of stirring sealed cultures (130 rpm) with liquid/air volume ratio of about 1:1.5. The plots show the average and errors (standard deviation) of three replicates representative of two independent experiments. The pictures (on the right) show the control cultures containing methylene blue on the 20^th^ day. In all experiments, the cells were pre‐adapted at 0.1% carbon source.

Next, we compared Mtb growth in solid media (Fig. S1). Mtb growth on 0.1% lactate, pyruvate or glucose is similar, while 0.4% glucose appears to promote faster growth. The different growth phenotype at the highest concentration could be due to the accumulation of a metabolite that inhibits the growth in lactate and pyruvate or to the reduced oxygen exchange compared to the growth in aerated liquid culture. This latter possibility agrees with the observation that the oxidation of lactate and pyruvate is reduced compared to glucose in limited aeration (Fig. 3C).

### Limited aeration affects the growth on lactate and pyruvate

In the recent Billig *et al*. study (Billig *et al.*, 2017), the authors claimed that lactate is a better carbon source than glucose until 20 mM, but it is toxic at 40 mM for H37Rv Mtb. This contrasts with our results, since at 44 mM (0.4%) Mtb H37Rv reaches higher OD than at 22 mM (0.2%) (Fig. 2B, E and F). This apparent discrepancy could be due to different growth conditions. Two important parameters that affect growth are agitation and liquid/air volume ratio. Our Mtb growth experiments were carried out in 100 ml of 7H9‐like media, in 1 L roller bottles, rolling at 2 rpm. Billig *et al*. grew Mtb H37Rv in minimal medium at 70 rpm, without specifying the culture volume used and the flask capacity. Finally, the length of the experiment described by Billig *et al.* (2017) was much shorter, and therefore not directly comparable to the data presented here.

To understand the potential role of aeration on lactate and pyruvate metabolism, we analysed the growth of Mtb H37Rv in glucose, lactate and pyruvate using different rolling speeds and liquid/air volume ratios. At 30 rpm and liquid/air volume ratio of about 1:5 (Fig. 3A) higher biomass was produced with increasing concentrations of lactate and pyruvate, but not glucose. In standing cultures with liquid/air volume ratio of about 1:10, an opposite trend was observed (Fig. 3B). In both experiments, lactate and pyruvate resulted in superior growth compared with glucose, and it seems that the aeration stimulates growth in lactate and pyruvate more strongly.

Closer inspection of the growth data in lactate at 0.4% (45 mM) at 2 rpm (Fig. 2B) and 30 rpm (Fig. 3A) reveals an initial growth delay compared to pyruvate and glucose, compatible with the length of the experiment carried out by Billig *et al.* This growth delay is also compatible with the slower growth observed in solid media at 0.4% (Fig. S1) and suggests that growth delay may be caused by differences in oxygen tension and lactate levels, rather than lactate toxicity.

We further tested Mtb growth in lactate, pyruvate or glucose using sealed tubes with slow stirring and a constant liquid/air volume ratio of 1:1.5, providing a slow decrease in aeration due to oxygen consumption by the bacteria (Wayne, 2001) (Fig. 3C). Although inspired by the, now classic, Wayne model of oxygen depletion, this experiment is unique as different carbon sources and different concentrations are used compared to conditions in the Wayne model. Surprisingly, under these conditions, glucose is the best carbon source (higher maximal OD). In fact, Mtb cultures containing lactate or pyruvate as carbon sources did not lead to bleaching of the methylene blue oxygen indicator (Fig. 3C). These results indicate that pyruvate and lactate cannot be utilised efficiently under low oxygen conditions.

### Metabolic network remodelling accompanies growth in lactate and pyruvate

To investigate in an unbiased fashion how Mtb responds to lactate and pyruvate, we compared transcriptomes and proteomes 24 h after cells were transferred from medium containing glucose and glycerol to medium containing lactate, pyruvate or glucose (reference condition) as principal carbon sources (Supplementary files 1 and 2).

**Figure 4:**
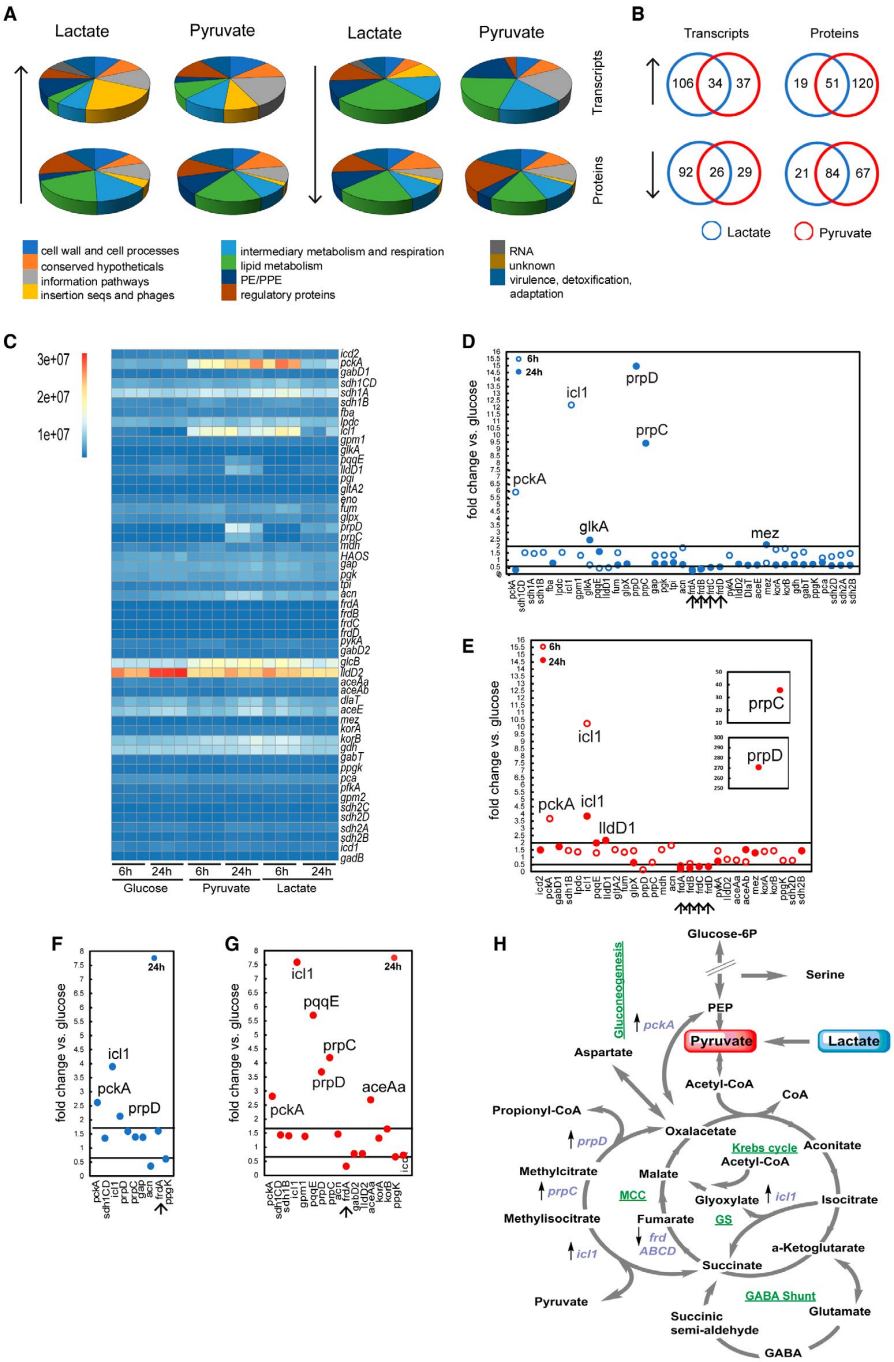
Transcriptomics and proteomics reveal that Mtb utilises the glyoxylate shunt and methylcitrate cycle to metabolise lactate and pyruvate. A. The pie charts show the percentage distribution in each functional category of transcripts (top panels) and proteins (bottom panels) significantly (↑) up‐ and or (↓) down‐regulated at 24 h. The distribution is calculated using the total gene number per category as ‘the 100%’ value. B. Venn diagrams showing comparison of (↑) up‐ and/or (↓) down‐regulated transcripts/proteins in lactate and pyruvate at 24 h. C. Heatmap showing the abundance (absolute reads) of central carbon metabolism (CCM) gene transcripts. Each column represents the reads abundance from one independent experiment at the indicated time point and carbon source. D–G. CCM transcripts (D) and proteins (F) fold‐changes in lactate; CCM transcripts (E) and proteins (G) fold‐changes in pyruvate; the lines across the charts represent the chosen fold‐change threshold. We considered significant differentially regulated those transcripts with a fold‐change ≥2 and ≤0.5, and those proteins with a fold‐change ≥1.7 and ≤0.6. We chose a lower threshold for protein fold‐change because the tandem mass tags (TMT) labelling methodology used in this work ‘compresses’ the fold‐change magnitude. The arrows at the bottom of the plots indicate significant down‐regulated genes both in lactate and pyruvate. Only the statistically significant fold‐change values were reported (*p* < 0.05). H. Schematic representation of CCM pathways; the differentially expressed genes both in lactate and pyruvate are reported in violet with the arrow direction indicating the up (↑) or down (↓) regulation. MCC = Methylcitrate Cycle. GS = Glyoxylate Shunt. Transcriptomics and proteomics results shown were obtained from three and two independent experiments respectively.

The analysis of functional categories of differentially regulated transcripts and proteins (Figs 4A, S2A and B) shows that growth in lactate or pyruvate induces global changes when compared to growth in glucose. Surprisingly, transcripts and proteins identifying with lipid metabolism category represent the largest number with altered regulation in each analysis, indicating significant remodelling of lipid metabolism. Notably, the absence of a perfect overlap (40–60%) in transcripts and protein levels between lactate and pyruvate (Figs 4B, S2C and Supplementary file 3) indicates that Mtb responds differently to these two highly similar carbon sources.

Only a few transcripts and proteins from the central carbon metabolism (CCM, Fig. 1) were differentially regulated, and interestingly, most of them showed the same regulation trend (up‐ or downregulation), in lactate and pyruvate. *pckA (*phosphoenolpyruvate carboxykinase*)*, *icl1 (*isocitrate lyase*), prpC* (methylcitrate synthase) and *prpD (*methylcitrate dehydratase*)* transcripts and corresponding proteins were strongly up‐regulated; whereas the *frdABCD (*fumarate reductase*)* operon and FrdA were down‐regulated (Fig. 4C and D–G). PrpR, the positive regulator of *prpDC* operon (Masiewicz *et al.*, 2012), was also up‐regulated at both RNA and protein levels, in both pyruvate and lactate (Supplementary files 1 and 2). The robust induction of these genes argues that Mtb employs the glyoxylate shunt and methylcitrate cycle to degrade lactate and pyruvate and *pckA* is likely used to channel oxaloacetate into gluconeogenesis (Fig. 4H). In pyruvate but not in lactate, there is an up‐regulation of AceAa (Fig. 4G), a paralogue of the *bona fide* isocitrate lyase, which displays low lyase activity for isocitrate (Honer Zu Bentrup *et al.*, 1999). Conversely, in lactate but not in pyruvate, *glkA* (glucokinase) and *mez* (malic enzyme) transcripts are up‐regulated (Fig. 4D).

The induction of methylcitrate cycle enzymes and transcriptional activator was unexpected. In bacteria, the methylcitrate cycle is considered a detoxification pathway responsible for the degradation of propionate, which is generated during catabolism of certain lipids (Dolan *et al.*, 2018). In Mtb, propionyl‐CoA is thought to be derived from β‐oxidation of odd‐chain fatty acids, degradation of host cholesterol and catabolism of branched amino acids. Importantly, the media used in this study did not contain cholesterol, fatty acids or branched‐chain amino acid that would require this pathway for metabolism.

Mtb has two annotated lactate dehydrogenase‐encoding genes, *lldD1*/*Rv0694* and *lldD2/Rv1872c*. Lactate dehydrogenase activity has been confirmed for LldD2, but not for LldD1 (Billig *et al.*, 2017). Surprisingly, we did not observe up‐regulation of *lldD2* in lactate compared to glucose conditions. Inspection of the raw data revealed that the *lldD2* transcript is the most abundant of the transcripts linked to CCM (Fig. 4C) and in the entire transcriptome (data not shown), in all carbon sources tested. *lldD1* is part of the *Rv0691A–Rv0694* transcriptional unit which has been suggested to be involved in the biosynthesis of a novel electron carrier molecule for Mtb, mycofactocin (Haft, 2011). We observed up‐regulation of *lldD1* transcript and *ppqE*/*Rv0693* protein (Fig. 4E and G) only in pyruvate. These results suggest that *lldD2* is constitutively expressed in Mtb and highlight a potential function for *lldD1* in pyruvate metabolism.

### Glyoxylate shunt is required during growth in lactate and pyruvate

**Figure 5:**
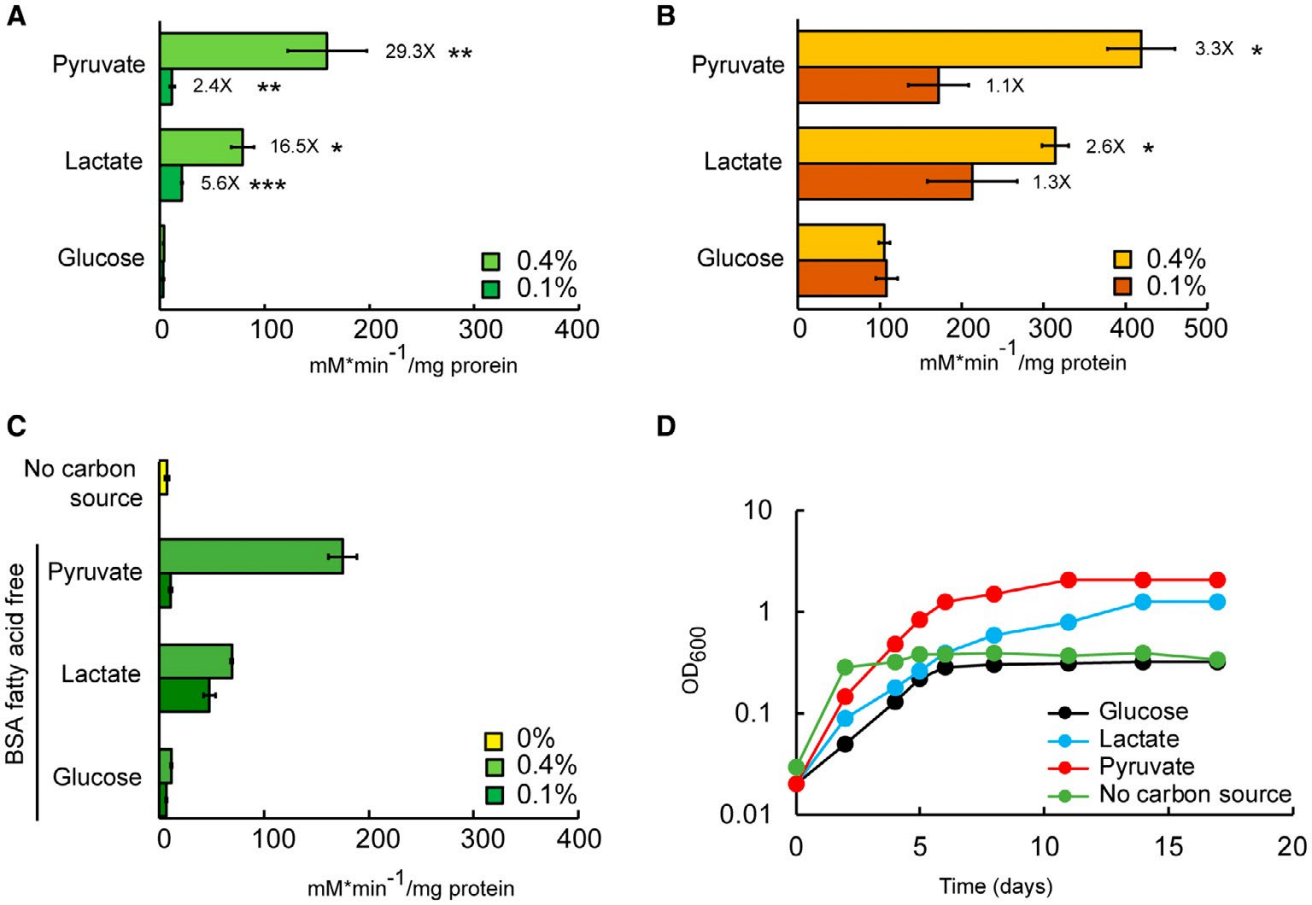
High levels of isocitrate lyase activity are present during the growth in lactate and pyruvate. A and B. Enzymatic activity of isocitrate lyase (A) and isocitrate dehydrogenase (B) measured in cell extracts of Mtb grown in lactate, pyruvate, or glucose as principal carbon source. Data represent the average and standard deviation from three independent experiments and two technical replicates for isocitrate lyase activity, and two independent experiments and two technical replicates for isocitrate dehydrogenase activity. The values at the top of the bars represent the fold‐change of the enzymatic activity detected in lactate or pyruvate vs the activity detected in glucose (minimum activity in pyruvate/lactate over maximum activity in glucose). *p value <0.00001; **p value <0.0003; ***p value <0.0002. C. Isocitrate lyase activity in cell extracts of Mtb grown in lactate, pyruvate and glucose as principal carbon source with BSA fatty acid‐free, and in medium with standard BSA but without the addition of carbon source. The data represent the average and error (average deviation) of two biological replicates. D. Growth curve in lactate, pyruvate or glucose as principal carbon source in medium supplemented with BSA fatty acid‐free, and in medium supplemented with standard BSA but no addition of carbon source.

Based on the transcriptomic and proteomic results, we wondered if the induction of *icl1* gene resulted in an actual increase in isocitrate lyase activity in Mtb during the growth in lactate or pyruvate. To verify this, cell‐free protein extracts from Mtb grown in glucose, lactate or pyruvate as principal carbon source were prepared for enzymatic activity measurements (Fig. 5). In agreement with the transcriptomic and proteomics data, significantly higher isocitrate lyase activity is observed in protein extracts from Mtb grown in lactate or pyruvate, compared to glucose (Fig. 5A). An additional increase in activity is observed at highest carbon source concentration, 0.4%. Next, we investigated if the activation of the glyoxylate shunt could lead to a down‐regulation of carbon flux through the Krebs cycle, despite the lack of down‐regulation of the isocitrate dehydrogenase (Icd) genes. Isocitrate dehydrogenase activity is similar at 0.1%, in all carbon sources, whereas the activity increases at 0.4% in extracts from lactate‐ or pyruvate‐grown Mtb, but not from glucose (Fig. 5B). These results indicate that an increased flux through the glyoxylate shunt is not accompanied by a decrease in flux through the Krebs cycle, but rather by an increase in flux at the highest concentration tested. Importantly, isocitrate lyase is also able to cleave methylisocitrate, an intermediate of the methylcitrate cycle, and therefore increased isocitrate lyase activity will likely result in increased methylisocitrate lyase activity.

Bovine serum albumin (BSA), present in 7H9 media, is known to contain bound fatty acids, which can be used as a carbon source by Mtb. To test whether the increase in isoitrate lyase activity was due to pyruvate or lactate and not due to the adventitious fatty acids from BSA, we repeated the experiment employing fatty acid‐free BSA (Fig. 5C). As an additional control, isocitrate lyase activity was measured in extracts derived from Mtb ‘grown’ in medium containing standard BSA, but no carbon sources. We observe that lactate and pyruvate carbon sources still induce higher levels of isocitrate lyase activity compared to the control conditions, indicating that small amounts of adventitious fatty acids present in BSA do not contribute to isocitrate lyase activation. In agreement with these results, growth in lactate and pyruvate is still superior compared to growth in glucose, when fatty acid‐free BSA is employed (Fig. 5D).

### Complex metabolism of lactate and pyruvate in Mtb

**Figure 6:**
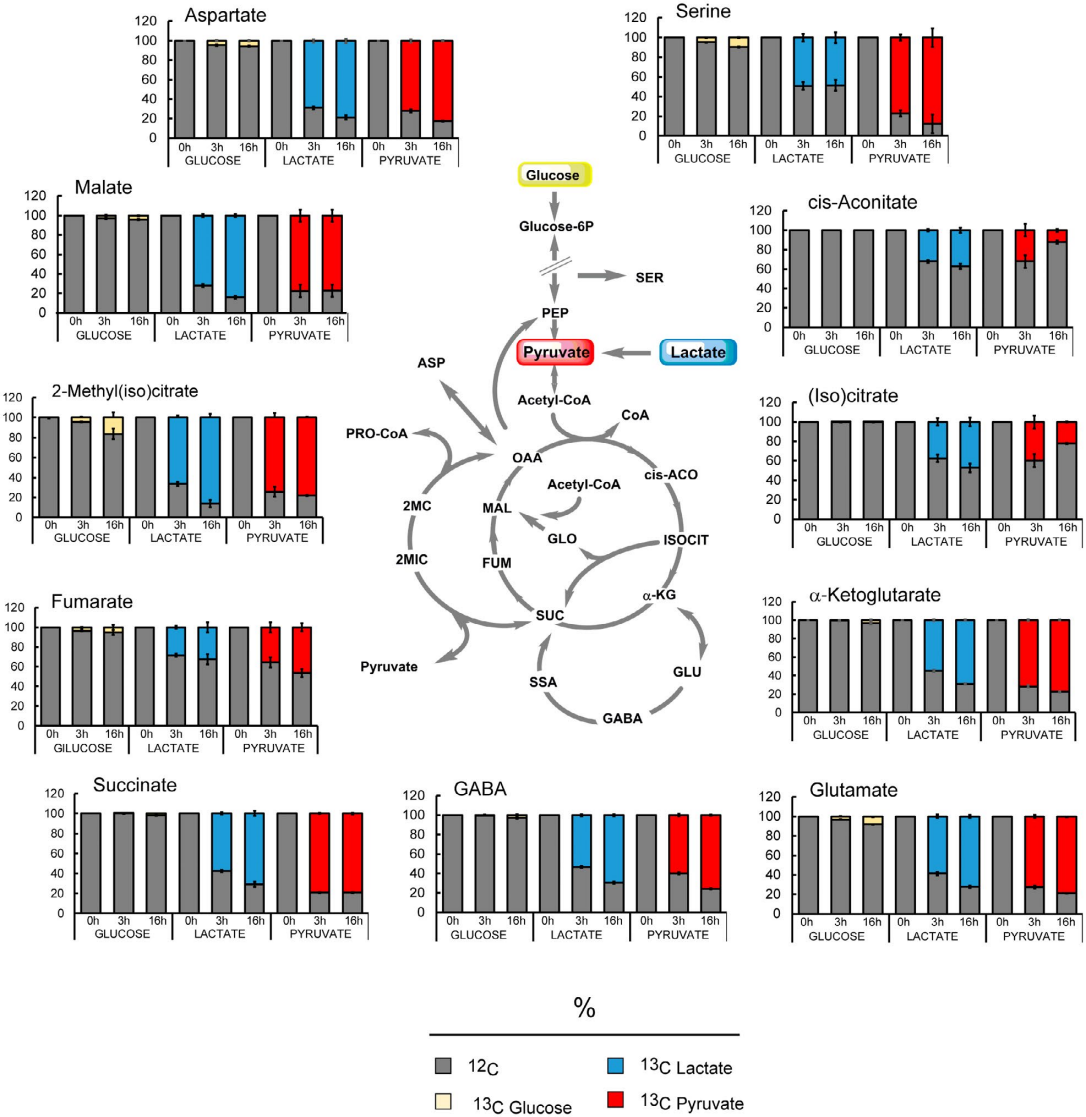
Metabolomics reveals that Mtb uses the entire CCM network to metabolise lactate and pyruvate. Percentage of labelled (^13^C) and unlabelled (^12^C) metabolites detected in Mtb grown in glucose, lactate or pyruvate at the concentration of 0.4%. 0h represents the unlabelled sample extracted from cells grown 6 days in unlabelled (U‐^12^C) carbon source. ‘3 h’ and ’16 h’ represent the exposure time to labelled carbon source. The charts show the average and standard deviation of four biological replicates from one experiment, representative of three independent experiments. cis‐ACO = cis‐aconitate; ASP = aspartate; FUM = fumarate; GABA = gamma aminobutyric acid; GLO = glyoxylate; GLU = glutamate; α‐KG = α‐ketoglutarate; ISOCIT = isocitrate; MAL = malate; 2MC = 2‐methylcitrate; 2MIC = 2‐methyl‐isocitrate; OAA = oxaloacetate; PEP = phosphoenolpyruvate; PRO‐CoA = propionyl‐CoA; SER = serine; SSA = succinic semialdehyde; SUC = succinate.

We used liquid chromatography–accurate mass Time‐of‐Flight (ToF) mass spectrometry metabolomics to track labelling of metabolites from CCM using uniformly (U)^13^C‐labelled carbon sources (Fig. 6). Carbon source pre‐adapted Mtb was exposed to (U)^13^C‐glucose, (U)^13^C‐lactate or (U)^13^C‐pyruvate for 3 h and 16 h. Although we did not observe significant changes in CCM metabolite pool size between lactate, pyruvate and glucose conditions (data not shown), even at 3 h post shift to labelled carbon source, CCM metabolites were readily labelled. Significant labelling in all detected metabolites was observed in cells grown in lactate or pyruvate as principal carbon source. In contrast, and as previously observed (de Carvalho *et al.*, 2010), labelling of intermediates of the Krebs cycle in cells grown in glucose as principal carbon source was negligible. These results indicate that both lactate and pyruvate are efficiently metabolised via the Krebs cycle, methylcitrate cycle, GABA shunt and gluconeogenesis (Fig. 6). The reduced labelling of fumarate compared to succinate may be compatible with down‐regulation of fumarate reductase (Fig. 4D and F) and suggests a preferential channelling of succinate into the methylcitrate cycle. The prominent labelling of L‐malate compared to fumarate likely derives from the glyoxylate shunt, and potentially from the malic enzyme (Mez) (Basu *et al.*, 2018), converting pyruvate to L‐malate. Notably, the reduced labelling of aconitate is incompatible with the vast labelling of the α‐ketoglutarate pool. There are at least two possible explanations for this result: i) aconitate might be partially derived from unlabelled metabolites; ii) α‐ketoglutarate labelling might be derived from alternative routes/stores, for example, from the GABA shunt or α‐ketoglutarate ferredoxin oxidoreductase (Baughn *et al.*, 2009). The incorporation of pyruvate‐ and lactate‐derived carbons in methyl(iso)citrate confirms the unexpected finding that Mtb uses methylcitrate cycle to metabolise these non‐lipidic carbon sources.

**Figure 7:**
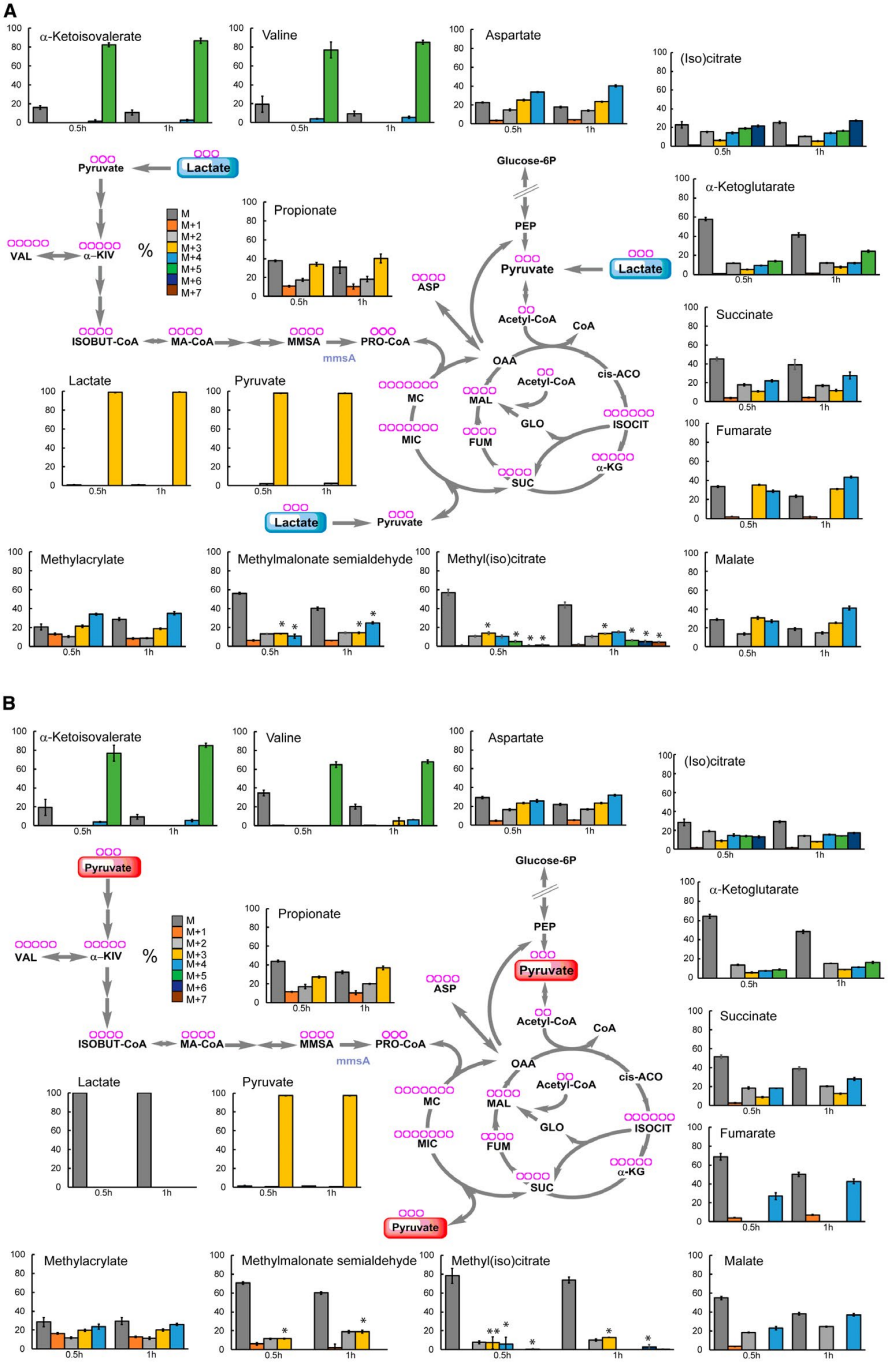
Isotopic species analysis reveals production of propionyl‐CoA by different pathways. Percentage of all the isotopic species in Krebs cycle, methylcitrate cycle, glyoxylate shunt and valine metabolism intermediates detected in Mtb grown in lactate (A) and pyruvate (B) at the concentration of 0.4%. The charts show the average and standard deviation of four biological replicates from one experiment, representative of two independent experiments. The purple rings at the top of ech metabolite name represent the number of carbon atoms. *p value <0.00001 between propionate M + 3 and the indicated isotopic species of methyl(iso)citrate and methylmalonate semialdehyde. cis‐ACO = cis‐aconitate; ASP = aspartate; FUM = fumarate; GLO = glyoxylate; α‐KG = α‐ketoglutarate; ISOBUT‐CoA = isobutyryl‐CoA; ISOCIT = isocitrate; MAL = malate; 2MC = 2‐methyl‐citrate; 2MIC = 2methyl‐isocitrate; MA‐CoA = methylacryl‐CoA; MMSA = methylmalonyl semialdehyde; α‐KIV = α‐ketoisovalerate; OAA = oxaloacetate; PEP = phosphoenolpyruvate; PRO‐CoA = propionyl‐CoA; SER = serine; SUC = succinate; VAL = valine.

To better understand the operation of these metabolic pathways with lactate and pyruvate, we analysed the individual isotopic species (isotopologues) of intermediates of the Krebs cycle, glyoxylate shunt and methylcitrate cycle at early time points, 0.5 and 1 h (Figs 7 and S3).

This analysis produced several confirmatory as well as novel results. (i) The qualitative labelling profiles of (iso)citrate, α‐ketoglutarate and succinate are compatible with the flux of pyruvate/lactate‐derived ^13^C through the oxidative branch of the Krebs cycle. (ii) The total ^13^C incorporation in (iso)citrate is higher compared to α‐ketoglutarate and succinate, and the total of ^13^C in succinate is higher compared to α‐ketoglutarate. These results support the interpretation that succinate labelling derives in part from isocitrate lyase. (iii) Fumarate labelling profile is not compatible with the succinate labelling. The lack of fumarate M + 2 species (Fig. 7) coupled with no changes in its pool size (not shown) suggests that fumarate is no longer being formed and consumed, and therefore succinate must be channelled elsewhere (e.g., in the methycitrate pathway). This result is confirmed by fumarate labelling profile at later time points (Fig. S4), while most of the isotopic species are present in succinate, only the M + 1 species is present in fumarate. Consequently, the presence of M + 2 species in malate (Fig. 7) suggests that this comes from glyoxylate shunt, via malate synthase. No M + 3 malate is observed in pyruvate (Fig. 7B) further supporting that malate is mainly produced by glyoxylate shunt when pyruvate is present as principal carbon source. (iv) The labelling profile of methyl(iso)citrate is compatible with the incorporation of succinate‐derived and pyruvate‐derived M + 2, M + 3, M + 4 species (Fig. S5). (v) The isotopic distribution in methyl(iso)citrate and propionate do not exactly match. Methyl(iso)citrate is labelled to a lower extent, compared to propionate (Fig. S3A), and M + 3 propionate abundance is higher than methylcitrate M + 3, M + 5, M + 6 and M + 7 species in lactate (Fig. 7A) and pyruvate (Fig. 7B). These results suggest that propionate is not solely produced via the methylcitrate cycle, even though pool sizes are similar with glucose, lactate or pyruvate as carbon source (Fig. S3B). (vi) The labelling profile of methyl(iso)citrate does not match with propionate M + 1 and M + 2 species. These results again suggest that propionate, derived from propionyl‐CoA, are synthesised by at least one more route.

Propionate and propionyl‐CoA could be derived from pre‐existing odd/branched chain fatty acid and/or branched chain amino acid catabolism; however, these intermediates would carry less or no label in this case. Fatty acid biosynthesis followed by catabolism would be necessary for labelling of core metabolic intermediates, but the length of these processes is inconsistent with a significant labelling of propionate observed after 0.5 h exposure to labelled lactate and pyruvate (Fig. 7). We next considered whether branched chain amino acid metabolism (de novo biosynthesis of valine and isoleucine, followed by degradation) could be providing part of the labelled propionate observed. Labelling of isoleucine was uninterpretable under our experimental conditions (see methods), and isoleucine catabolism intermediates were not detected. In addition, no changes in genes/proteins involved in isoleucine metabolism are present in our transcriptomics and proteomics data. In contrast, valine metabolism was clear (Figs 7, S3B and S4). Valine biosynthesis starts with the condensation of two molecules of pyruvate and, as expected, the heaviest isotopic species (M + 5) of valine and its direct precursor/first degradation product (α‐ketoisovalerate), was the most abundant (80%) (Figs 7 and S4). Looking at degradation intermediates, such as methylacrylate and methylmalonate semialdehyde, we also noticed a significant labelling of these metabolites. In contrast, their isotopic distribution matched better with the isotopic distribution found in propionate than with the one observed in valine and α‐ketoisovalerate. In addition, between 0.5 h and 1 h an increase in the abundance of M + 3 propionate was observed (Fig. 7). This result matches with an increase in abundance of the heaviest isotopologues of methylacrylate and methylmalonate semialdehyde. The utilisation of valine biosynthesis/degradation for the synthesis of propionyl‐CoA is also supported by the observed up‐regulation of *mmsA* transcript and protein, in pyruvate (Supplementary file 1 and 2). *mmsA* encodes a methylmalonate semialdehyde dehydrogenase that converts methylmalonate semialdehyde and NAD^+^ into propionyl‐CoA, HCO_3_^‐^ and NADH. However, the labelling profiles of these intermediates suggest that they derive from another route rather than valine/α‐ketoisovalerate degradation alone.

Altogether, these results confirm the activation of methylcitrate cycle to metabolise lactate and pyruvate as carbon sources, revealing reversal of the cycle as an unprecedented route for carbon assimilation and propionyl‐CoA production in Mtb. Moreover, we discover that another pathway, involving MmsA, also appears to be involved in the formation of propionyl‐CoA in Mtb.

### *pckA* and *icl* genes are essential for the growth of Mtb in pyruvate and lactate

**Figure 8:**
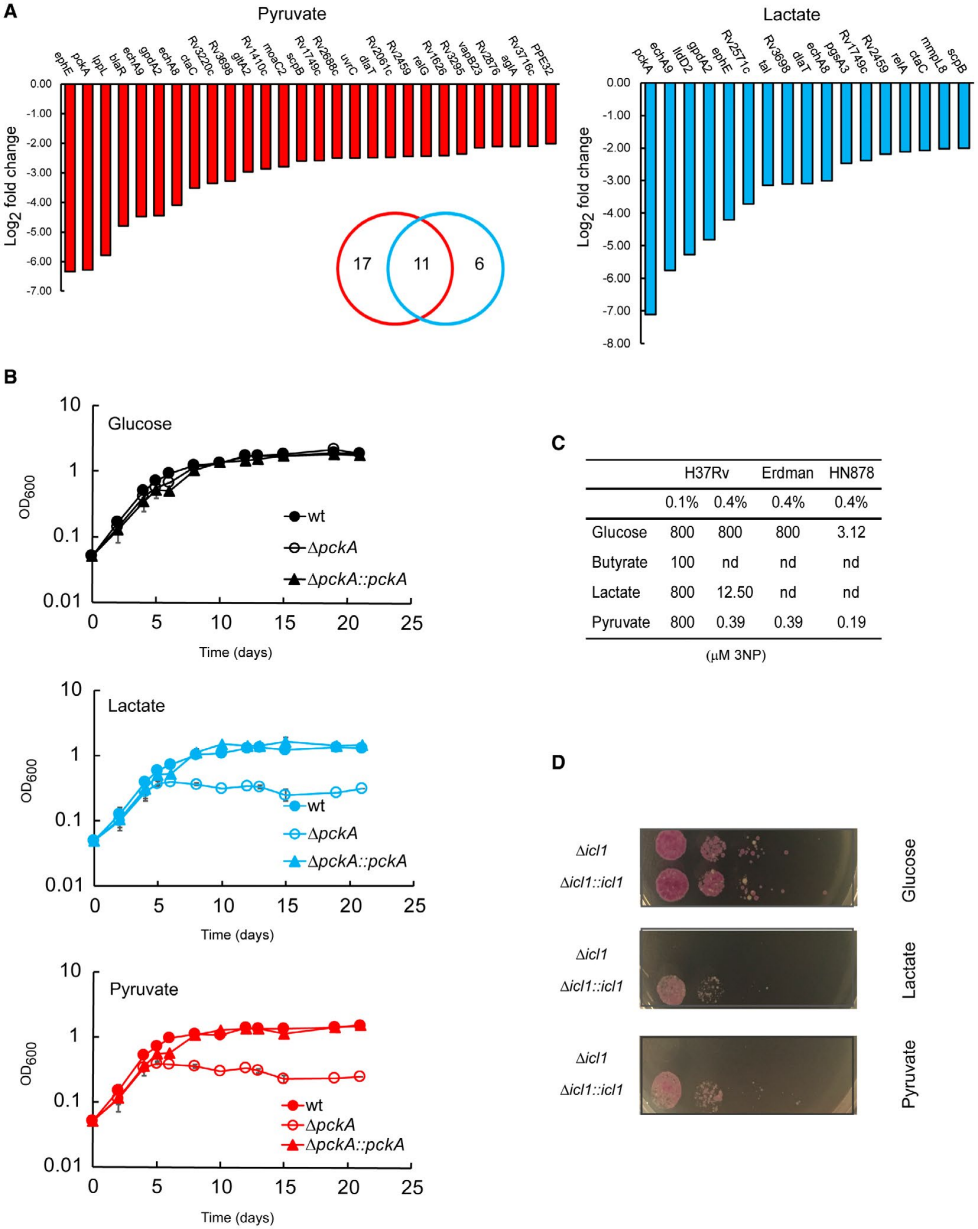
Phosphoenolpyruvate carboxykinase and isocitrate lyase are essential for lactate and pyruvate utilisation. A. Growth reduction of transposon mutants in pyruvate and lactate (0.4% concentration). The fold change is calculated vs glucose + glycerol (0.2% + 0.2% concentration). The Venn diagram shows the number of mutants with reduced growth only in lactate (blue ring), only in pyruvate (red ring) and in both (ring intersection). B. Growth of *pckA* knockout mutant and its parental strains (Erdman and complemented mutant) in 0.1% of carbon source. The charts report the average and error (average deviation) of two independent experiments representative of three. The growth observed during the first 4 days is likely due to intracellular glucose stored in the form of glucose phosphate, trehalose or glycogen. C. MIC of 3‐nitropropionate (μM) estimated in solid media at different carbon source concentrations in three Mtb strains. The values are representative of two independent experiments. D. Growth of an H37Rv *icl1* mutant strain and its complemented in solid media at 0.4% of carbon source. 5 µl of serial dilutions of 0.1% carbon source pre‐adapted cultures were spotted on the media. The pictures were taken after 25 days of growth and are representative of two independent experiments. The pink colour is due to the presence of a replicative plasmid expressing hsp60′::mCherry in both strains.

To identify genes required for growth on lactate or pyruvate, we performed a transposon mutant library screen using medium containing 0.2% glucose and 0.2% glycerol as a reference condition. From this screening, we identified several mutants with reduced growth in lactate or pyruvate (Fig. 8A and supplementary file 4). *dlaT* (E2 subunit of the pyruvate dehydrogenase and α‐ketoglutarate dehydrogenase complexes) and *gltA2* (citrate synthase) mutants were identified, confirming that some pyruvate and lactate are directed to the Krebs cycle/glyoxylate shunt. As expected, *lldD2* (lactate dehydrogenase) absence is detrimental to growth in lactate, but not in pyruvate. *pckA* is the only gene/transcript/protein that was identified by all three platforms, i.e., transcriptomics, proteomics and TraDIS, in both lactate and pyruvate. To unambiguously demonstrate the role of PckA in pyruvate and lactate metabolism in Mtb, we examined the growth of a clean *pckA* knock‐out (KO), parent (Mtb Erdman) and complemented strains in lactate and pyruvate. The *pckA* KO strain is unable to grow in 0.1% (Fig. 8B) and 0.4% (data not shown) lactate or pyruvate, as principal carbon sources. As expected, no growth defect is observed in 0.1% (Fig. 8B) and 0.4% (data not shown) glucose. While this work was in progress, the importance of *pckA* activity in lactate utilisation was revealed by Billig *et al.* (2017).

Although highly induced, *icl1* and *prpDC* genes were not identified as genes required for growth in pyruvate or lactate in the TraDIS screening, suggesting that they may not be essential for lactate and pyruvate metabolism or in the case of *icl1* because of the redundancy afforded by *aceAa*. Additionally, the medium used as reference in the TraDIS screen contained glycerol which is partially dissimilated by Mtb via isocitrate lyase, for degradation of glycerol‐derived pyruvate (Beste *et al.*, 2011). Therefore, to assess the importance of isocitrate lyase and methylcitrate cycle enzymes for the growth in lactate and pyruvate, we analysed Mtb growth in the presence of a potent isocitrate lyase inhibitor, 3‐nitropropionate (3NP) (Honer Zu Bentrup *et al.*, 1999), using butyrate as a positive control of inhibition (Early *et al.*, 2016) and characterised growth profile of a *prpDprpR* mutant (Δ*MCC*). At lower carbon source concentration (0.1%), Mtb growth was only modestly affected by 3NP in lactate, pyruvate or glucose compared to butyrate. At 0.4% carbon source concentration, 3NP appears to be a potent inhibitor of Mtb growth in lactate and pyruvate, but not in glucose (Figs 8C and S6a). Similar results were obtained in liquid media for lactate, while a partial inhibition was observed in pyruvate (Fig. S6B). 3NP can also inhibit succinate dehydrogenase (Alston *et al.*, 1977), albeit at higher concentrations. To provide independent evidence of the importance of isocitrate lyase for lactate and pyruvate metabolism, we analysed the growth of a H37Rv *icl1* mutant. In agreement with the results obtained using 3NP inhibitor, the absence of isocitrate lyase activity prevents the Mtb growth in lactate and pyruvate in solid media (Fig. 8D) and also in pre‐adapted liquid culture for lactate (Fig. S6C). Together, these results demonstrate that isocitrate lyase is required for Mtb growth in lactate and pyruvate at 0.4%. While this work was in progress, the importance of *icl* activity in pyruvate growth was showed using an Erdman *icl* double mutant strain (Baker and Abramovitch, 2018). The different phenotypes in pyruvate‐containing liquid and solid media might be due to lower isocitrate lyase activity levels in solid cultures compared to liquid cultures. The isocitrate lyase activity may be affected by multiple factors such as growth phase, oxygen tension and the different exposure to nutrients, which vary between solid and liquid cultures.

Finally, we analysed the growth of a *prpRprpD* mutant and its parental strain (Erdman background). In contrast to *icl1*, *prpR* and *prpD* are not essential for lactate and pyruvate growth with or without pre‐adaptation in these carbon sources (data not shown and Fig. S6D).

### Differential utilisation of lactate but not pyruvate by Mtb strains

**Figure 9:**
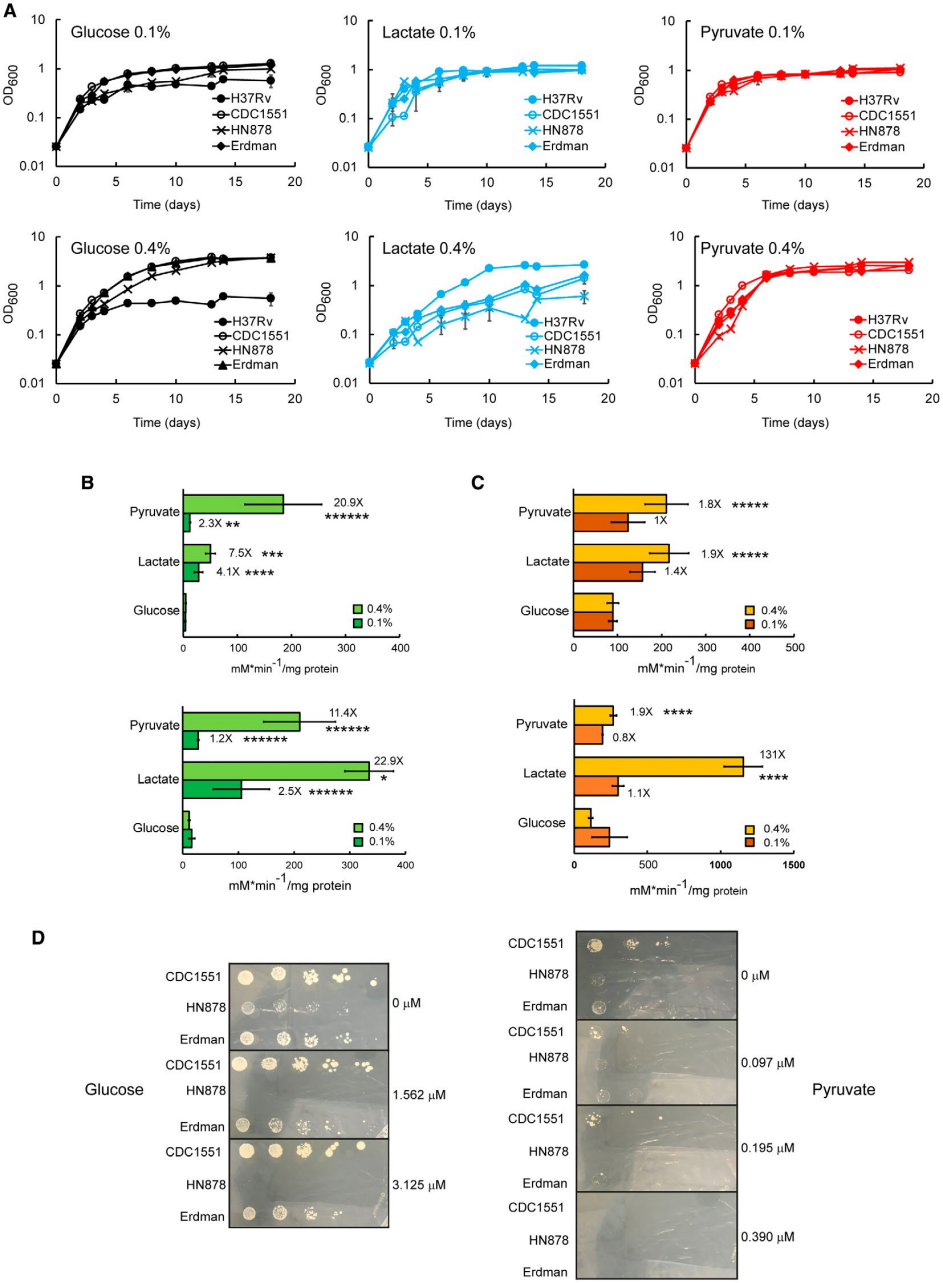
The assimilation of lactate and pyruvate is similar between H37Rv, CDC1551, Erdman and HN878 strains. A. Growth in lactate, in pyruvate or in glucose as carbon sources. The plots show the average and error (average deviation) of two independent experiments. B and C. Enzymatic activity of isocitrate lyase (B) and isocitrate dehydrogenase (C) measured in cell extracts of Erdman (top panels) and HN878 (bottom panels) grown in lactate, in pyruvate or in glucose as one carbon source. The plots show the average and error (average deviation) of two independent experiments and two technical replicates. The values at the top of the bars represent the fold changes of the activity detected in lactate or pyruvate vs the activity detected in glucose (minimum activity in pyruvate/lactate over maximum activity in glucose). *p value <0.00001; **p value <0.0003; ***p value <0.0002; ****p value <0.003; *****p value <0.008; ******p value <0.05. D. Growth of Mtb strains on solid media supplemented with the specific carbon source and different concentration of 3NP (µM). 5 µl of serial dilutions of 0.1% and 0.4% carbon source pre‐adapted cultures were spotted on the media. The pictures were taken after 30 days of growth, and they are representative of two independent experiments.

During the examination of *pckA* essentiality in lactate and pyruvate as sole carbon sources, we noticed that Mtb Erdman strain (the parent strain used to construct the pckA KO strain) behaved differently with respect to Mtb H37Rv strain, during the growth in lactate and glucose. Intrigued by these different phenotypes, we extended our growth analysis to other Mtb strains, including HN878 and CDC1551 (Sreevatsan *et al.*, 1997; Plikaytis *et al.*, 1999). At 0.1% carbon source all Mtb strains tested grow similarly in lactate, pyruvate or glucose (Fig. S9A, top panels). Of note, H37Rv reaches lower OD in glucose, compared to the other strains. Interestingly, at 0.4% carbon source, Erdman, CDC1551 and HN878 strains reached much higher cell density in glucose and conversely, display growth delay in lactate, compared to H37Rv (Fig. 9A, bottom panels). Together, these results suggest that: (i) H37Rv stands slightly apart from the others Mtb strains, with regards to glucose and lactate metabolism; (ii) all Mtb strains tested appear to use pyruvate as principal carbon source efficiently; and (iii) there is an inverse correlation between superior growth in glucose and in lactate. Importantly, the chief role of *pckA* gene in Erdman and H37Rv backgrounds (Fig. 8A and B) suggests that pyruvate and lactate metabolism mediated by PckA is not strain dependent.

Subsequently, we investigated whether isocitrate lyase and isocitrate dehydrogenase activity increased in these strains during the growth in lactate and in pyruvate, as carbon sources. As we observed with H37Rv strain lysates (Fig. 5A), lysates from Erdman and HN878 strains show higher isocitrate lyase activity in lactate and in pyruvate, compared to glucose (Fig. 9B). Isocitrate dehydrogenase activity increases at 0.4% pyruvate or lactate in Erdman, whereas in HN878 there is a dramatic (131‐fold) increase of isocitrate dehydrogenase activity when cells were cultured in lactate, but not in pyruvate (Fig. 9C).

Next, we tested the effect of 3NP on different Mtb strains cultured in pyruvate and confirmed the same H37Rv MIC value for Erdman and a fold lower value for HN878 (Figs 8D and 9D). Surprisingly, 3NP inhibits HN878 growth in glucose. This result is supported by the higher isocitrate lyase activity measured in lysates from HN878 cells grown in glucose, compare to Erdman and H37Rv (Fig. 9B) and indicates that HN878 relies more on isocitrate lyase (i.e., the glyoxylate shunt, and possibly the methylcitrate cycle) to grow in glucose as main carbon source. These results show that the growth inhibition by 3NP in pyruvate, and likely lactate, is not strain dependent.

### Abundant lactate and pyruvate inside human macrophages

**Figure 10:**
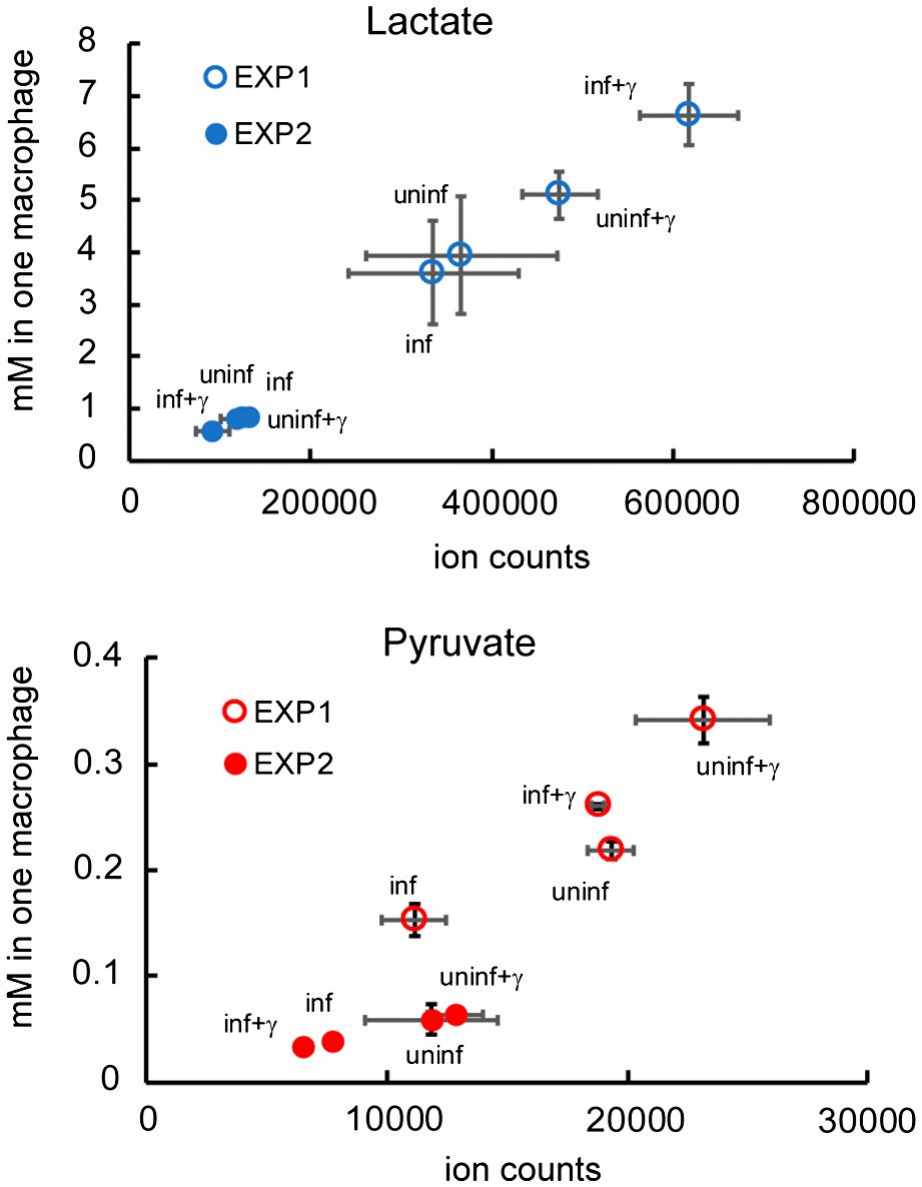
Concentrations of lactate and pyruvate in one infected macrophage. Lactate and pyruvate concentration in one macrophage plotted against the detected ion counts. The results from two independent experiments are individually displayed. Each dot in the chart shows the average and standard deviation of four biological replicates.

Pyruvate and lactate are present in several human cells as physiological by‐products of glucose catabolism. Macrophages are well‐known to be highly glycolytic and to reduce glycolysis‐generated pyruvate to lactate. Lactate produced by macrophages, as well as by many other cells, is thought to be rapidly exported by specific transporters. If the concentration of lactate inside macrophages is negligible, it would not serve as a physiologic carbon source. In contrast, it is known that Mtb can utilise lactate as a sole carbon source *in vitro* (Youmans and Youmans, 1953; Billig *et al.*, 2017), and this work). To the best of our knowledge, the concentration of pyruvate and lactate in macrophages, and therefore the possibility that it can be used as an intracellular carbon source by Mtb, is not known. To estimate the concentration of lactate and pyruvate in macrophages, we measured lactate and pyruvate from infected human‐derived macrophages (3 × 10^6^ cells) and culture media (extracellular), by LC‐MS. Although the results from two independent experiments were slightly different (Figs 10 and S7), as the macrophages were obtained from two different donors, metabolomic analysis consistently detected lactate and pyruvate from the macrophage metabolome, i.e., intracellular (Figs 10, S7A and B). In agreement with current knowledge of macrophage physiology, the amount of secreted lactate is much higher than the pool of intracellular lactate (Fig. S7B and C). Using lactate and pyruvate concentrations obtained from 1 ml of macrophage extract (from 3 × 10^6^ cells), we calculated the approximate concentration of lactate and pyruvate per macrophage (Fig. 10), using as reference the average volume of one J774 macrophage (Melmed *et al.*, 1981; Krombach *et al.*, 1997). We estimated that lactate varies from 0.56 to 6.7 mM and pyruvate varied from 0.03 to 0.34 mM, inside macrophages. Therefore, our data on human macrophages suggests that both lactate and pyruvate are abundant carbon sources available inside macrophages, suggesting that lactate and pyruvate could be used by Mtb intracellularly as well extracellularly.

## Discussion

Our study demonstrates that Mtb uses several metabolic pathways (the glyoxylate and GABA shunts, the Krebs and methylcitrate cycles, valine metabolism and gluconeogenesis) to metabolise lactate and pyruvate. In particular, we demonstrate that glyoxylate shunt and the methylcitrate cycle, pathways traditionally associated with fatty acid utilisation in Mtb (Munoz‐Elias and McKinney, 2005; Munoz‐Elias *et al.*, 2006; Gould *et al.*, 2006; Marrero *et al.*, 2010), are also employed for lactate and pyruvate metabolism. Additionally, we demonstrated the importance of isocitrate lyase and pyruvate carboxykinase activities for the growth both in lactate and pyruvate. These results lead us to conclude that the previously demonstrated inability of Mtb *pckA* (Marrero *et al.*, 2010) and *icl* (Munoz‐Elias and McKinney, 2005) mutants to establish a productive infection may be due to a defect in lactate and pyruvate metabolism, in addition to an inability to utilise lipids as carbon sources.

Interestingly, our growth experiments under define carbon source conditions indicate that Mtb can grow until concentrations of lactate and pyruvate as high as 40 mM, achieving higher growth rates and higher biomass, compared to fatty acids. Based on these results, it is tempting to speculate that high micromolar to millimolar levels of highly soluble terminal glycolytic products such as pyruvate and lactate might be more suitable carbon sources compared to lipids. In addition to fatty acids, cholesterol has been suggested as a carbon source used by Mtb during infection (Pandey and Sassetti, 2008) but the role of cholesterol as an important carbon source *in vivo* has recently been challenged, as the *hsd* gene, encoding the first enzyme in cholesterol catabolism, has been shown to be dispensable for Mtb replication in guinea pigs (Yang *et al.*, 2011). Moreover, Mtb appears to replicate without attenuation in lipid droplet‐deficient mice (Knight *et al.*, 2018), suggesting that alternative carbon sources such as lactate and pyruvate might be available to Mtb as preferred substrates.

Recently, lactate utilisation by Mtb was suggested to be important during infection. Indeed, a lactate dehydrogenase mutant is not able to replicate in human macrophages (Billig *et al.*, 2017). Pyruvate is converted to lactate in macrophages, and lactate is rapidly exported. To understand if lactate and pyruvate are present at levels sufficiently high to enable intracellular Mtb growth, we evaluated lactate and pyruvate concentrations in macrophages (Fig. 10). We estimated that in blood‐derived human macrophages intracellular lactate concentration varied from 0.56 to 6.70 mM and pyruvate concentration varied from 0.03 to 0.34 mM. These results support the idea that both lactate and pyruvate are abundant intracellular carbon sources in human macrophages. These measurements are of course not perfect, as they do not take into account compartmentalisation, metabolite distribution, the actual volume of macrophages or changes in metabolism during infection *in vivo*, and therefore, it is possible that Mtb experiences higher or lower concentrations of lactate and pyruvate during infection. Mtb can replicate extracellularly and in necrotic human macrophages (Lerner *et al.*, 2017), where lactate and pyruvate concentrations might reach 44 mM. The fact that Mtb can grow very well at these concentrations suggests that these substrates may be necessary and utilised by Mtb at certain stages of infection. It is known that the MCT4 lactate transporter is present in the phagosomal membrane of *Mycobacterium bovis* bacille Calmette–Guerin‐infected macrophages (Lee *et al.*, 2010) raising the possibility that lactate may be available during the intraphagosomal stage. Of note, MCT4 is a bidirectional, acidic pH‐stimulated transporter (Sasaki *et al.*, 2014), which suggests that it might be active at the phagosomal membrane.

**Figure 11:**
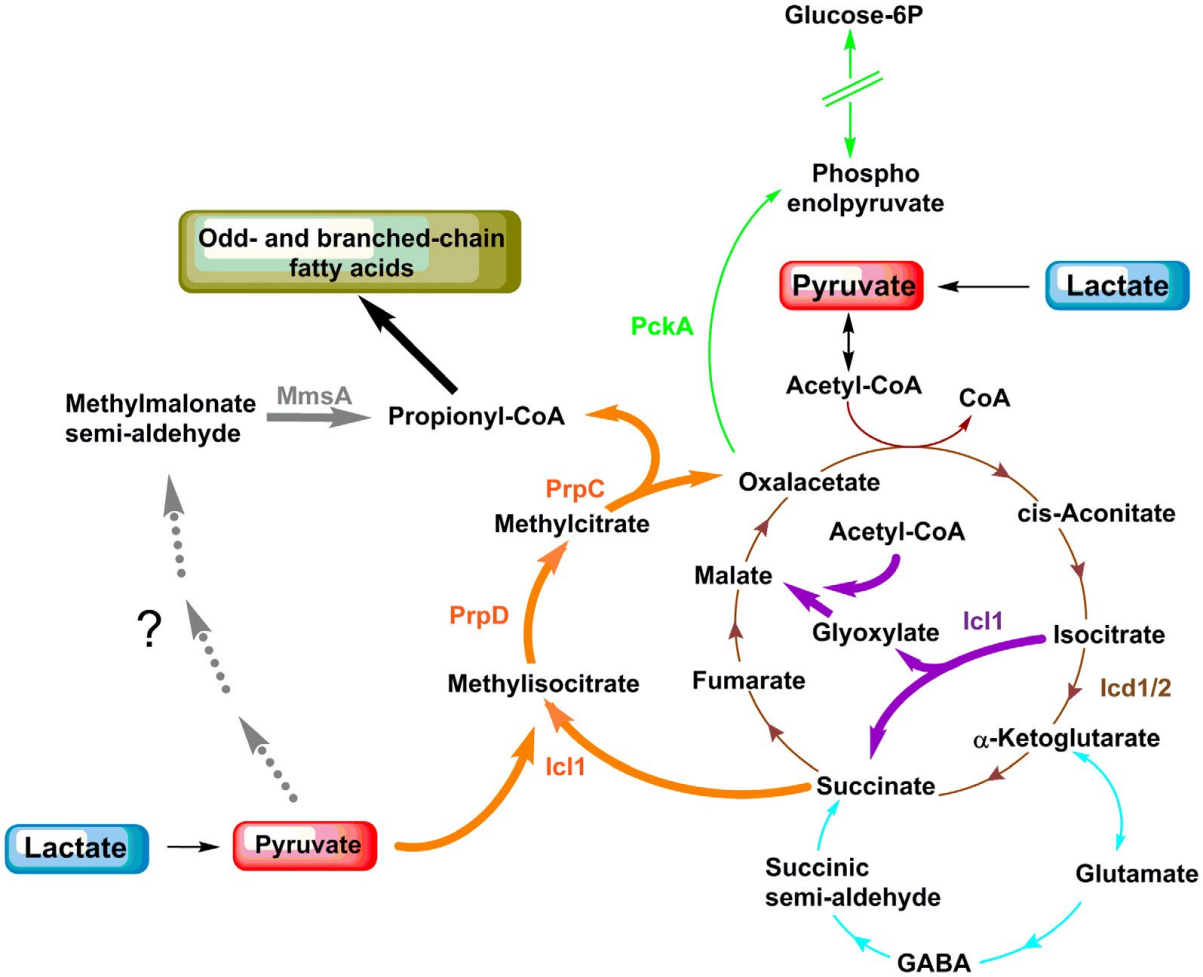
Metabolic network utilised for assimilation of lactate and pyruvate in Mtb. The scheme illustrates the pathways identified in this study that Mtb employs to metabolise lactate and pyruvate; gluconeogenesis (green), Krebs cycle (brown), glyoxylate shunt (violet), methylcitrate pathway (orange), GABA shunt (light blue) and a novel uncharacterised pathway, which includes MmsA enzyme, to synthesise propionyl‐CoA (grey). Bold arrows are used to underline the main pathways. The enzymes involved in lactate and pyruvate metabolism which were identified in this study are highlighted with the colours of the respective pathways they belong to.

An unexpected finding of our study is the operation of the methylcitrate cycle in reverse, with lactate and pyruvate. Of note, no source of propionate was present in these experiments, and our data (Figs 6, 7, S3 and S4) suggests that the propionate present in the cells does not derive from the degradation of pre‐existing endogenous lipids and branched chain amino acids. Given the carbon sources provided and the labelling profiles observed, sole operation in the canonical direction is inconsistent with the data. Interestingly, our results indicate that, together with methylcitrate cycle, another pathway is active to produce propionyl‐CoA from lactate and pyruvate, which seems to involve the methylmalonate dehydrogenase (*mmsA*) (Fig. 11).

Propionyl‐CoA is a substrate for odd‐chain fatty acid biosynthesis (Dolan *et al.*, 2018) and this fate is consistent with a differential expression of several genes encoding enzymes involved in lipid metabolism (Figs 4A and S2A, B). Surprisingly, among those, the only genes that have been functionally characterised are the ones involved in lipid biosynthesis (Supplementary file 5). Our TraDIS results also link lipid metabolism to lactate and pyruvate metabolism (Supplementary file 5). The most well‐characterised hit on TraDIS is *mmpl8*, encoding an exporter involved in the biosynthesis and export of type I sulfolipid (SL‐1) (Converse *et al.*, 2003; Domenech *et al.*, 2004). In contrast, *PapA*1 and *Pks*2 (Seeliger *et al.*, 2012) transcripts and proteins (essential enzymes involved in SL‐1 biosynthesis) are down‐regulated in lactate and pyruvate, compared to glucose. With a role for Mmpl8 in SL‐1 export being detected during Mtb growth in glucose and glycerol as carbon sources, we suggest that SL‐1 might not be the main sulfolipid produced during growth in lactate or pyruvate. Mtb can also produce SL‐2 and SL‐3 (Rhoades *et al.*, 2011; Sarpe *et al.*, 2016), and Mmpl8 might, therefore, transport different sulfolipids. It will be interesting to investigate, in a comprehensive manner, the cell envelope lipid composition of Mtb grown in different carbon sources.

Mtb may experience hypoxic environments in granulomas (Tsai *et al.*, 2006) and lactate production by the host increases during hypoxia (Roiniotis *et al.*, 2009). In this work, we have demonstrated that limited aeration negatively affects Mtb growth in lactate and pyruvate (Fig. 3) and we observed that different Mtb strains display altered growth at high lactate concentrations (Figs 2, S1 and 9). These phenotypes strongly suggest Mtb activates specific physiological responses to different oxygen and carbon source concentration. Further investigations are necessary to address if a functional axis exists between high lactate and pyruvate concentrations and low oxygen levels, which might control Mtb persistence in intra‐ and extracellular hypoxic environments.

Finally, effectors of ESX‐1 secretion system (EsxA and EsxB), essential for Mtb virulence (Houben *et al.*, 2014), are differentially regulated in lactate and pyruvate compared to glucose. A filamentous structure formed by EspC (Lou *et al.*, 2017) mediates the secretion of EsxA, which exhibits phagosome membrane lysing activity, allowing Mtb to escape into the cytosol (de Jonge *et al.*, 2007) during infection. We observed down‐regulation of EspC and its chaperone, EspA, transcripts and proteins both in lactate and in pyruvate; and up‐regulation of EsxA and its chaperone, EsxB, proteins but not of their transcripts (Supplementary files 1 and 2). The greater abundance of EsxA and EsxB proteins may be due to blocked secretion caused by reduced *espACD* expression. These results imply the existence of a connection between ESX‐1 activity and carbon metabolism, which has not been described before. Considering these findings, it would be interesting to investigate additional links between virulence factors and bacterial metabolism.

## Conclusion

Fig. 11 summarises the parts of Mtb's metabolic network employed to assimilate lactate and pyruvate, revealed by this study. The most striking result from our investigation is the requirement of glyoxylate shunt and reverse methylcitrate cycle for lactate and pyruvate metabolism. It seems that these pathways originally identified as ‘lipid‐specific’ are in fact more generally used by Mtb to cope with metabolic‐challenging environments. Reverse methylcitrate cycle provides a direct and short route for the biosynthesis of propionyl‐CoA, which is the essential precursor for the biosynthesis of odd‐ and branched chain fatty acids, abundantly found in Mtb cell envelope (Layre *et al.*, 2011). Additionally, we discovered that Mtb has another short route for biosynthesis of propionyl‐CoA revealing that this metabolite may have a crucial role in Mtb physiology. Mtb's cell envelope composition is a key virulence factor, and redundant pathways producing lipid precursors can guarantee its complete and continue effectiveness during infection. A recent genome‐wide association study of clinical isolates (Hicks *et al.*, 2018) revealed that *prpR* mutations confer multidrug tolerance. Our results lead us to hypothesise that this multidrug tolerance may be the consequence of a reduced drug permeability due to an alteration of odd‐ and branched chain fatty acid composition of cell envelope lipids.

This study furthers our understanding of how terminal glycolytic intermediates serve as carbon sources for Mtb. Our results reveal the full extent of involvement of core metabolic pathways in lactate and pyruvate metabolism. Furthermore, as several mutants lacking enzymes important for lactate and pyruvate metabolism have already shown a significant attenuation in the macrophage and mouse models of infection (McKinney *et al.*, 2000; Munoz‐Elias and McKinney, 2005; Munoz‐Elias *et al.*, 2006; Marrero *et al.*, 2010), our results point to a re‐interpretation of the mechanistic role and utilisation of these pathways by Mtb during infection, i.e., their potential importance in lactate and pyruvate metabolism for Mtb *in vivo*.

## Experimental procedures

### Strains and growth condition

Mtb H37Rv strain was employed in all experiments. Mtb CDC1551, Erdman and HN878 strains were used to investigate strain specificity of metabolic phenotypes. The *pckA* knockout strain (Erdman genomic background), its parental and its complemented strains was kindly provided by Sabine Ehrt (Weill Cornell Medicine) (Marrero *et al.*, 2010). The *icl1* knockout strain have been described elsewhere (Lee *et al.*, 2013). It contains a replicative plasmid expressing hsp60′::mCherry and for this reason the colonies appear dark pink.

The strains were routinely grown in 10 ml of medium in 50‐ml Falcon tubes at 30 rpm in 7H9 (BD Difco) supplemented with 0.5% bovine serum albumin 0.2% glucose, 0.85% NaCl, 0.05% tyloxapol and 0.2% glycerol (ADNTG). All the experiments comparing carbon sources were performed in 7H9 or 7H10 supplemented with 0.5% bovine serum albumin, 0.85% NaCl, 0.05% tyloxapol and the specific carbon source selected between glucose (D‐glucose), lactate (L‐lactate), pyruvate, valerate, butyrate and oleate at the concentration indicated in the figures. Liquid growth experiments were performed using 100 ml of medium in 1 L roller bottle rolling at 2 rpm, unless stated otherwise. For growth experiments with the *pckA, icl1* and *prpRprpD* knockout strains (and their parental) in oleate, the cells were pre‐adapted in the specific carbon source for 4–5 days using the lowest concentrations used in this study.

For transcriptomic and proteomics experiments, H37Rv was grown in 100 ml of 7H9 supplemented with ADNTG in roller bottles. When the culture reached OD_600_ = 0.5–0.7, the cells were harvested and washed 3 times with 7H9 without supplement. Then, they were suspended in 100 ml of fresh 7H9 supplemented with the specified carbon source at the final concentration of 0.4%. The samples were collected after 6 h and 24 h for RNA extraction and at 24 h for protein extraction.

Erdman *prpRprpD* (*ΔMCC*) mutant strain were generated by allelic exchange as previously described (Mann *et al.*, 2011). The *ΔMCC* mutant was created by inserting a hygromycin resistance cassette into the chromosomal locus that encodes the methylcitrate cycle enzymes PrpD and PrpC. The region that was replaced by allelic exchange in this mutant spanned 344 bp of the 5′ end of rv1129/prpR and the entire rv1130/prpD sequence. This mutation removes the *prpD and prpR* promoters and the *prpD* gene. Allelic exchange was confirmed by sequencing and Southern analysis using the Direct Nucleic acid Labelling And Detection Kit (GE Health Care).

The *Δicl1* strain was complemented cloning a region containing about 500bp upstream of the annotated start codon and the entire ORF in the pKP203 vector (Rossi *et al.*, 2011).

### RNA extraction, cDNA library and sequencing

Thirty millilitres of bacterial suspension were centrifuged at 3000 g for 5 min and the pellet was quickly frozen in dry ice and stored at −80°C until the extraction. The frozen pellet was slowly thawed on ice. Total RNA was extracted according to the FastRNA Pro Blue kit manual (MP). The cells were lysed by bead‐beating using a FastPrep‐24 (MP Biomedicals) 3 times (40 s/6.0), incubating on ice for 1 min between pulses. To remove genomic DNA, the samples were treated twice with TurboDNase (Ambion) for 1 h at 37°C. Total RNA was purified and concentrated to a final volume of 20 µl using DEPC water and the RNA clean‐up kit (Zymoresearch). Approximately 2.5 ng of RNA was used to check the removal of DNA by PCR (Fw TTTGTTTGGAGAGTTTGATCCTG and Rv CTCATCCCACACCGCTAAA primers specific for RNA16S gene). The nucleic acid integrity was verified by Bioanalyzer (Agilent RNA 6000 Nano).

Five micrograms of each RNA samples were depleted of rRNA using the Ribo‐Zero Magnetic Kit (Epicentre). The samples were purified using Qiagen RNeasy MinElute Cleanup Kit by means of modified protocol suggested in the Ribo‐Zero Magnetic Kit to allow small RNAs recovering. RNA was quantified by Qubit. The library was prepared using ScriptSeqv2 RNA‐Seq kit (Epicentre), FailSafe Enzyme Mix Only and ScriptSeq Index PCR Primers Set1#RSBC10948 (Epicentre) according to the manual. The library was sequenced with an Illumina Hi‐Seq 4000 instrument.

### RNA‐seq data analysis

Paired‐end fastq files for each sample were aligned to the H37Rv reference sequence using Bowtie2 (Langmead and Salzberg, 2012) retaining the default parameters; in particular, zero mismatches allowed in the seed alignment, no read‐clipping and a maximum fragment length of 500 bases. Raw alignment scores for coding exons and cnRNAs were determined using samtools bedcov (0.1.9) (Li *et al.*, 2009). Normalisation and differential expression calling were performed using the DESeq2 package (Love *et al.*, 2014) in R 3.3.1 (The R Core Team ‘R: A Language and Environment for Statistical Computing’ (2016) https://www.R-project.org/). Heatmaps (Fig. S8) were generated using the pheatmap package also in R (Kolde, R. ‘pheatmap:Pretty Heatmaps’, 2015, https://CRAN.R-project.org/package=pheatmap). For the purposes of presentation only, ‘outlier’ counts were identified by pair‐wise regression of within condition replicates (Fig. S9). Points for which the Studentized residuals in one or more regression were in excess of 20, or for which the positioning was in the upper left region of the first quadrant (specifically: X ≤ 2,000,000 & Y ≥ 5,000,000) were defined to be outliers and were excluded from presentation in the heatmap. Figures S8 and S9 show the quality and reproducibility of the experiments.

### Protein extraction and proteolysis

Protein extraction was performed using a modified protocol described in Schubert *et al.*, 2015 (Schubert *et al.*, 2015). Fifty millilitres of bacterial suspension were centrifuged at 3000 g for 10 min. The pellet was washed 3 times with cold Dulbecco's phosphate‐buffered saline (DPBS) 1X, quickly frozen in dry ice and stored at −80°C until the extraction. The cells were defrosted on ice and 1 ml of lysis buffer (0.1 M TEAB buffer, 8 M Urea, 0.1% Rapigest, Complete EDTA‐free) was added. The suspension was thoroughly vortexed and incubated at RT for 10′ (with gently shaking). Cells were lysed by bead‐beating using a FastPrep‐24 (MP Biomedicals) for three 40 s cycles (6.0 set; 2:1 = buffer volume: beads volume). After each cycle, the samples were incubated on ice for 1 min and centrifuged at 13,000 rpm for 10 min and fresh lysis buffer was added to the beads for the next cycle. In total, 1.7 ml of lysis buffer were used for each sample. The extracts were filtered twice using PVDF‐filtered tubes (10 min at 12,000 g) before removal from BLS3. The protein content was measured using the Pierce BCA Protein Assay Kit. One milligram of protein was subjected to proteolysis. Protein disulphide bonds were reduced by adding DTT to a final concentration of 10 mM and incubating for 30 min at 30°C; then the free cysteine residues were alkylated by adding freshly made iodoacetamide to a final concentration of 20 mM for 45 min at RT in the dark. Again, 10 mM DTT was added (i.e., the total DTT added is 20 mM including the step 1 aliquot), and the samples were incubated for 30 min at 30°C. 1:100 w/w LysC (Wako) was added to each sample and incubated at 30°C for 4 h. Because the high concentration of urea can inhibit trypsin activity, the samples were diluted with 0.1 M TEAB to <2 M urea concentration. Then, 10 µg (1:100, w/w) of sequencing grade‐modified trypsin (Promega) were added and the samples were incubated at 30°C overnight. The complete digestion was checked loading 5 µg of digested extract on 12% acrylamide gel.

### TMT labelling and LC‐MS analysis

The proteome was analysed by mass spectrometry using Tandem Mass Tag (TMT) labelling for quantification. The peptides obtained from proteolytic digestion of protein extracts were purified using silica C18 microspin columns (NEST). The columns were conditioned with 50% acetonitrile and 0.5% acetic acid; 0.1% of trifluoroacetic acid (TFA) was used to desalt the samples (three washing steps). The samples were eluted in 50% acetonitrile and 0.5% acetic acid, dried in a vacuum centrifuge and then re‐suspended in 100 µl of 50 mM HEPES (pH 8.5)/30% (v/v) acetonitrile. 10plex TMT label reagents were added in each sample and the mix was incubated for 1 h at room temperature and quenched with 0.5% hydroxylamine. Prior to mixing, the individual TMT‐labelling efficiency was checked and found to be >95%. TMT‐labelled samples were mixed, dried in a vacuum centrifuge, re‐suspended in 300 μl 0.1% TFA and sonicated. The samples were fractionated using a High pH Reverse‐Phase Fractionation Kit (Thermo), dried, resuspended in 1% TFA and analysed on a Lumos Orbitrap (Thermo, Hemel Hempstead UK).

Data analysis (identification and quantification) was performed using MaxQuant software (Cox and Mann, 2008). The H37Rv2 database of Mtb sequences concatenated with common contaminants was used for peptide identification. Volcano plots were generated in Perseus with permutation‐based false discovery rate (FDR) of 0.05 to cater for multiple hypothesis testing. The principal component analysis from two independent experiments and two technical replicates is showed in Fig. 10A.

### Metabolite extraction

Cells were grown in 100 ml of 7H9 supplemented with ADNTG in roller bottles until OD_600_ of 1.0. Approximately 1 × 10^8^ cells were collected by filtration on 0.22‐µm mixed cellulose filters (Millipore) and transferred on 7H10 supplemented with 0.4% of glucose, lactate or pyruvate as one carbon source. In all 7H10 plates, tyloxapol was omitted to avoid interfering during liquid chromatography. Plates were incubated 6 days at 37°C to allow bacterial replication and produce biomass; four filters per condition were used as 0h control samples, then the other filters were transferred on fresh plates of 7H10 supplemented with 0.4% of uniformly (U)‐^13^C‐labelled glucose, lactate or pyruvate as sole carbon source. The polar metabolites were extracted in a pre‐chilled (−40°C) methanol:acetonitrile:water (2:2:1) solution and cells were lysed twice by bead‐beating using a FastPrep‐24 (MP Biomedicals) for 30 s (6.5 power set; 2:1 = buffer volume: beads volume) incubating on ice for 1 min between pulses. Soluble extracts were filtered (cellulose‐acetate filter tubes) at 17,000 g for 20 s at 4°C and stored at −80°C.

For metabolite extraction from macrophages, 250 μl supernatant was collected from each condition and mixed with three parts cold acetronitrile:methanol:water. Cells were then washed with PBS and lysed in 300 μl cold acetonitrile:methanol:water. Three wells (total of 3 x 10^6^ cells) were pooled for each technical replicate. Extracts were vigorously vortexed then centrifuged at 16,000 g for 20 min at 4°C. The supernatant was then transferred to cellulose filter tubes and centrifuged twice at 16,000 g for 20 min to remove residual bacteria and stored at −80°C for LC‐MS analysis.

### Metabolomics with liquid chromatography–mass spectrometry

The liquid chromatography–mass spectrometry was essentially performed as previously described (Larrouy‐Maumus *et al.*, 2016), with some modifications. Aqueous normal phase liquid chromatography was performed using an Agilent 1200 LC system at controlled temperature (4 ºC). Flow rate of 0.4 ml min^−1^ was used. Elution polar were performed using a gradient of two solvents, A (mQ water and 0.1% of formic acid) and B (acetonitrile and 0.1% of formic acid). Only in the experiment showed in Figure 4, the metabolites analysed in positive mode (aspartate, glutamate, GABA and serine) were eluted using 0.2% acetic acid instead of 0.1% formic acid.

The data were analysed by Profinder B.08.00 software and Masshunter Qualitative Analysis B07.00. The metabolites were identified comparing the accurate m/z (error < 10 ppm) and the retention time with the accurate m/z and the retention time of standard solutions for the specific metabolite. The percentage of labelling was calculated using the total ion counts for the specific metabolite in the specific sample as ‘100’ value. Then the average and the standard deviation between percentage values from four biological replicates were calculated and plotted. The abundance (pool size) was quantified using standards calibration curves, and then the concentration was normalised by the residual protein concentration (detected by BCA assay) present in each extract. To get an accurate and more tangible concentration of metabolites, the standard solutions for calibration curves were diluted in 1/3 of extracts mixture.

Glyoxylate levels were too low to be detected under our experimental conditions and oxaloacetate is intrinsically unstable (Rej, 1985). As an alternative to oxaloacetate, we measured aspartate. We cannot distinguish methylcitrate and methylisocitrate or leucine and isoleucine in our LC‐MS conditions. Serine was used to track the gluconeogenesis carbon flux. Principal component analysis for metabolomics data is showed in Fig. S10B and C.

### Enzymatic activity

Cells were grown in 7H9 supplemented with the selected carbon source until mid‐log phase. Fifty millilitres of bacterial suspension were centrifuged at 3000 g for 10 min. The pellet was washed 3 times with cold DPBS 1X. 1 ml of lysis buffer (DPBS 1X supplemented with complete EDTA free) was added. Cells were lysed twice by bead‐beating using a FastPrep‐24 (MP Biomedicals) for 40 s (6.0 set; 2:1 = buffer volume: beads volume) incubating on ice for 1 min between pulses. The extracts were centrifuged at 13,000 rpm for 10 min at 4°C, the supernatant was filtered with PVDF filter tube twice before removal from BLS3. Fifty microlitres of extract were used to perform the reaction. Isocitrate lyase activity: the reaction was performed following the protocol described in Munoz‐Elias *et al.*, 2006 (Munoz‐Elias and McKinney, 2005); isocitrate‐stimulated NADH oxidation was monitored. Isocitrate dehydrogenase activity: the reaction was performed following the protocol described in Tian *et al.*, 2005 (Tian *et al.*, 2005); we used NADP^+^ as co‐substrate; the NADP^+^ reduction was monitored. Both reactions were measured recording the absorbance variation for 8 min at 340 nm and using the standard extinction coefficient 6.22 mM for a 1 cm path length.

### 3‐nitropropionate MIC

The strains were pre‐adapted in 7H9 supplemented with one carbon source selected between glucose, lactate, pyruvate and butyrate at the same concentration used in the assay. Cultures at mid‐log phase were diluted at OD_600_ of 0.1 and 1:10 serial dilutions were performed in 7H9 without carbon source. Five microlitres of each dilution were spotted on 7H10 plates supplemented with the specific carbon source (0.1 or 0.4%). 3‐Nitropropionate (Sigma) was added at the concentration range from 0.024 to 800 µM. The inhibition in liquid culture was performed inoculating pre‐adapted cells (0.2% carbon source in 10 ml of 7H9 shaken at 30 rpm) at the final OD of 0.01 in 100 ml of 7H9 supplemented with the specific carbon source in 1‐L roller bottles at 2 rpm rolling.

### Screening of transposon mutant library and gDNA extraction

Tn*5* transposon mutant library of *Mtb* H37Rv was subjected to a screen to identify genes required for the utilisation of lactate or pyruvate as principal carbon source. Glycerol stocks of the mixed mutant library were first thawed and washed five times with 7H9 + 0.05% tyloxapol to remove traces of media components. The washed library was then diluted to an OD_600_ of 0.2 in 30 ml of each media (Control: 7H9 supplemented with ADNTG; Test condition: 7H9 supplemented with 0.4% lactate or pyruvate (see above for the medium composition) representing an average of 3x10^4^ CFU each, of approximately 10,000 Tn mutants, and grown at 37°C in roller bottles at 30 rpm. The cultures were grown to an OD_600_ of ~2, sub‐cultured (10‐fold) in their respective media to an OD_600_ of 0.2 and allowed to grow again to an OD_600_ of ~2. Cells were subsequently pelleted prior to gDNA extraction. Cell pellets were resuspended in 700 μl TE buffer and transferred to a screw‐cap tube containing 0.1 mm Zirconia/silica beads (0.3 volume). Cell lysis was performed via bead‐beating using the BeadBug^TM^ microtube homogenizer (Benchmark Scientific) at three pulses of 4000 rpm for 30 s, chilling on ice between each pulse. Beads and cell debris were pelleted, and the cell lysate was transferred to a fresh 2 ml microcentrifuge tube. An equal volume of phenol:chloroform:isoamyl alcohol (25:24:1) was added to the lysate and centrifuged. The top aqueous phase was retained and washed once more, before a final wash step with chloroform:isoamyl alcohol (24:1). The resultant aqueous layer was then treated with RNAse A (overnight 4°C) and genomic DNA was purified via ethanol precipitation. Precipitated genomic DNA were washed twice with ice‐cold 70% ethanol and air‐dried prior to being resuspended in 10 mM Tris.

### Transposon‐directed insertion site sequencing (TraDIS)

Genomic DNA from each sample (L‐lactate, pyruvate, control) was subjected to library preparation using the Nextera DNA Sample Prep kit (Illumina) following the manufacturer's instructions with modifications to amplify and sequence Tn*5* insertion sites. Briefly, genomic DNA was enzymatically fragmented and tagged with an adapter sequence in a single reaction (tagmentation). Following tagmentation, the DNA was purified using the Zymo DNA Clean and Concentrator kit (Zymo Research). This is followed by a PCR enrichment step performed using index primer 1 (one index per sample) and a custom transposon‐specific primer [5′‐AATGATACGGCGACCACCGAGATCTACACgcctgaagatcttctagactgcaggcatgcaag −3′ (transposon‐specific sequence is in lowercase)] to enrich for transposon insertion sites and allow multiplex sequencing (72°C for 3 min and 98°C for 30 s, followed by 22 cycles of 98°C for 10 s, 63°C for 30 s and 72°C for 1 min). The resultant library was purified using Agencourt Ampure XP magnetic beads. Library verification and quantification were performed via Qubit 2.0 fluorometer and the 4200 Tapestation system (Agilent Technologies). All libraries were pooled and submitted for sequencing on the Illumina MiSeq platform at the Australian Centre for Ecogenomics (University of Queensland, Australia). The MiSeq sequencer was loaded with 12 pM of pooled library with 5% PhiX spike‐in and sequenced (single‐end, 101 cycles) using a mixture of standard Illumina sequencing primer and Tn*5*‐specific sequencing primer (5′‐ CAGGCATGCAAGCTTCAGGGTTGAGATGTGTA‐3′).

### Analysis of TraDIS data

Analysis of TraDIS data was performed essentially as previously described, with some modifications (Goh *et al.*, 2017). Raw de‐multiplexed fastq files from the MiSeq run were filtered and trimmed to capture reads containing the Tn*5* specific tag (5′‐TAAGAGACAG‐3′) at their 5′ ends by using the FASTX toolkit (http://hannonlab.cshl.edu/fastx_toolkit/index.html). These reads were aligned to the H37Rv genome using Bowtie (version 1.1.2) (Langmead *et al.*, 2009) with its default arguments, and aligned reads were reported with ‘‐M 1 –best’ parameters. Subsequent analysis steps were carried out in ‘R’ (version 3.3.1), with the Rsamtools package (version 1.26.1) (Morgan *et al.*, 2018) to calculate the number of sequence reads (read counts) after normalisation of total reads for each sample. The number of reads per gene from pyruvate or L‐lactate samples were compared to reads from the control sample and expressed as log2 fold‐change (logFC) to identify genes significantly attenuated in the respective conditions. A logFC of >2 is considered significant.

## Author contributions

AS and LPSC designed the project; AS, LT, S Howell, DJG, DMH, MM, CRM carried out experiments; BV constructed the *prpRprpD* mutant strain; AS, LT, S Horswell, DMH, MM, BV, MDP, MS, AGG, APS, NPW, analysed data; AS, MGG, NPW, LPSC interpreted data; AS, S Horswell, LPSC prepared figures; AS and LPSC wrote and revised the manuscript, with inputs from the other authors.

## Data Availability

Transcriptomics raw data were deposited at the NCBI Genomic Expression Omnibus (GEO) platform under accession number GSE116859. Proteomics raw data were deposited at the PRoteomics Identification (PRIDE) archive under Project accession number PXD010726. The TraDIS raw sequence data were deposited on the Sequence Read Archive (SRA) under the Bio Project accession number PRJNA484103 and SRA accession number SRP156112 (https://www.ncbi.nlm.nih.gov/bioproject/PRJNA484103).
