# Supplementary material for: Mycobacterium tuberculosis requires glyoxylate shunt and reverse methylcitrate cycle for lactate and pyruvate metabolism
Source: Mol Microbiol. 2019 Aug 23;112(4):1284–307. doi: 10.1111/mmi.14362 (PMC6851703; doi:10.1111/mmi.14362)
Supplement: Supplementary file 6 [file MMI-112-1284-s006.docx]

**Supplementary Information**

***Mycobacterium tuberculosis* requires glyoxylate shunt and reverse methylcitrate cycle for lactate and pyruvate metabolism.**

Agnese Serafini^1^, Lendl Tan^2^, Stuart Horswell^3^, Steven Howell^4^, Daniel J. Greenwood^5^, Deborah Hunt^1^, Minh-Duy Phan^2^, Mark Schembri^2^, Mercedes Monteleone^6^, Christine R. Montague^7^, Warwick Britton^6^, Acely Garza-Garcia^1^, Abraham P. Snijders^4^, Brian VanderVen^7^, Maximiliano G. Gutierrez^5^, Nicholas P. West^2^ and Luiz Pedro S. de Carvalho^1*^

^1^Mycobacterial Metabolism and Antibiotic Research Laboratory, The Francis Crick Institute, 1 Midland Road, London, NW1 1AT, UK, ^2^ School of Chemistry and Molecular Biosciences, The University of Queensland, 4072, Australia. ^3^Bioinformatics and Biostatistics Science Technology Platform, ^4^Mass Spectrometry Science Technology Platform and ^5^Host-Pathogen Interactions in Tuberculosis Laboratory, The Francis Crick Institute, 1 Midland Road, London, NW1 1AT, UK, ^6^Mycobacterial Research Program, Centenary Institute of Cancer Medicine and Cell Biology, Camperdown NSW 2050, Australia ^7^Department of Microbiology and Immunology, College of Veterinary Medicine, Cornell University, Ithaca, United States.

**Running Title**: Metabolism of lactate and pyruvate in *Mycobacterium tuberculosis*

***** **Address for correspondence:**

Luiz Pedro S. de Carvalho, PhD

Laboratory of Mycobacterial Metabolism and Antibiotic Research
The Francis Crick Institute, London NW1 1AT, UK

[luiz.carvalho@crick.ac.uk](mailto:luiz.carvalho@crick.ac.uk)

+442037962300

**Supplementary Figure Legends**

**
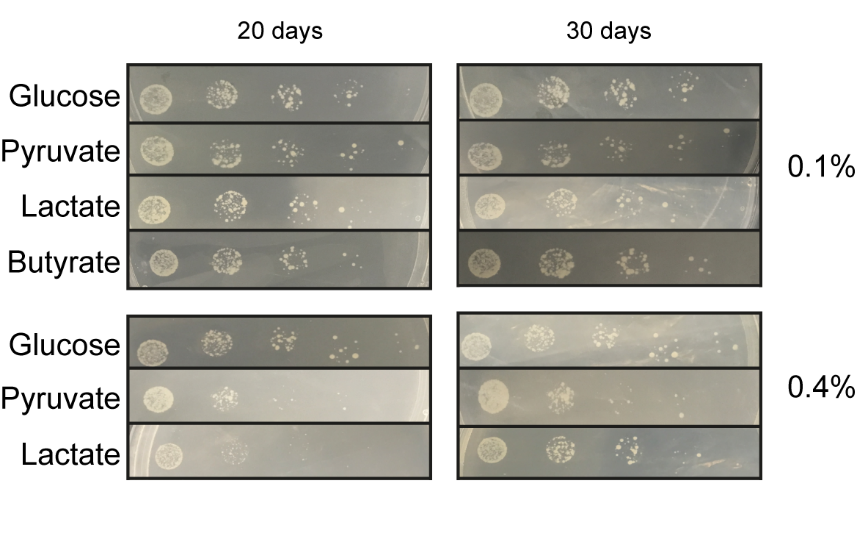
Figure S1**

**Figure S1. H37Rv solid media growth in lactate and pyruvate.** 5 µl of serial dilutions of 0.1 % carbon source pre-adapted cultures were spotted on solid media supplemented with the specific sole carbon source. Pictures after 20 and 30 days of growth were taken and they represent one of 2-3 independent experiments.

**Figure S2**

**
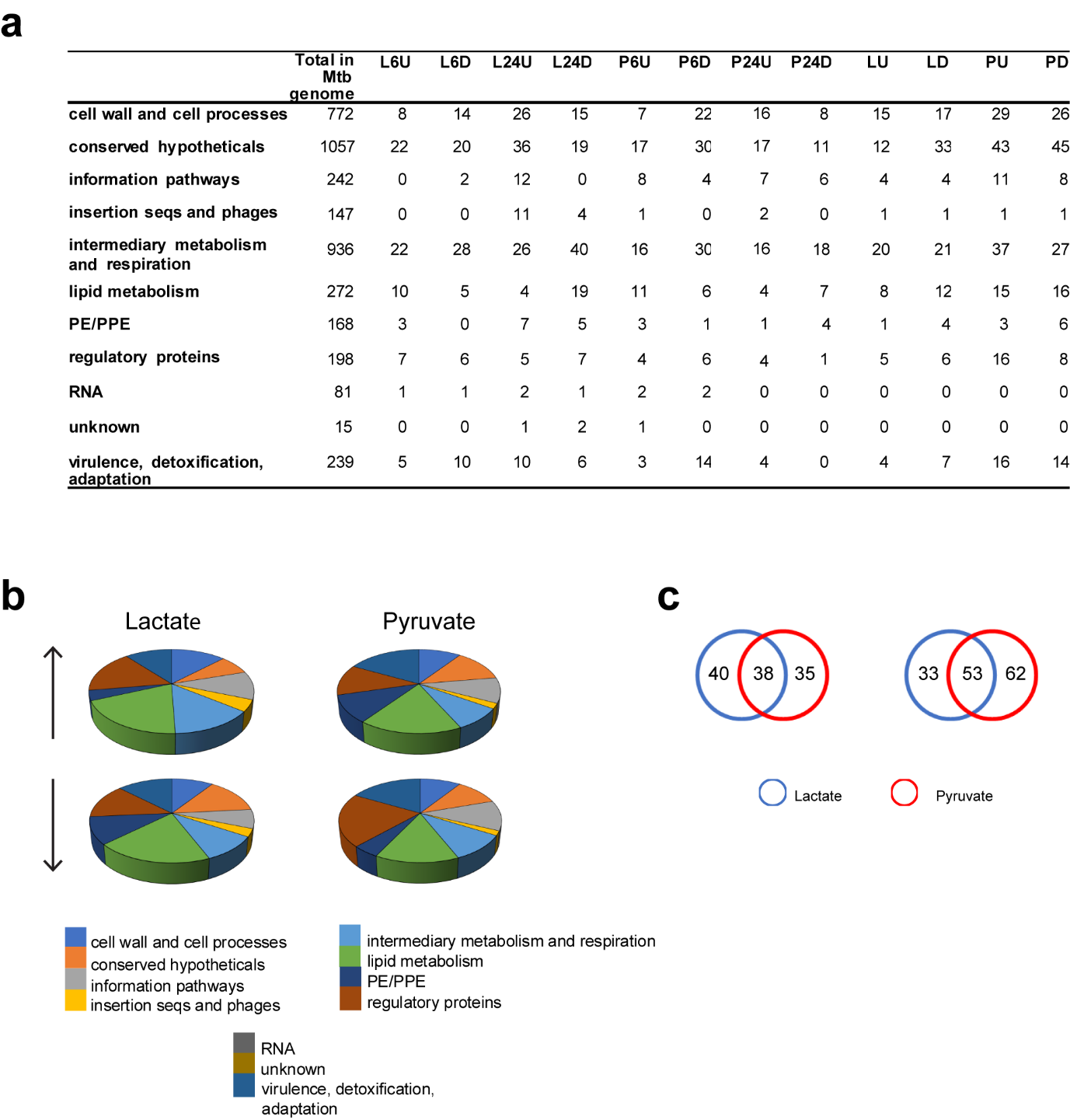
**

**Figure S2. Functional category distribution and comparison of differentially regulated transcripts and proteins between lactate and pyruvate**. **a**) The table lists the functional categories and the number of transcripts and proteins significantly differentially regulated per category. The “Total in Mtb genome” column lists the total number of Mtb genome genes per category. Transcripts. L6U: lactate up-regulated at 6h; L6D: lactate down-regulated at 6h; L24U: lactate up-regulated at 24h; L24D: lactate down-regulated at 24h; P6U: pyruvate up-regulated at 6h; P6D: pyruvate down-regulated at 6h; P24U: pyruvate up-regulated at 24h; P24D: pyruvate down-regulated at 24h. Proteins. LU= lactate up-regulated; LD= lactate down-regulated; PU= pyruvate up-regulated; PD= pyruvate down-regulated. **b**) The pie charts show the percentage distribution in each functional category of transcripts significantly up (top) and down (bottom)-regulated at 6h in pyruvate and lactate. The percentage is calculated using the total number of genes per category (“Total in Mtb genome” column in the table) as “the 100 %” value. **c**) Venn diagrams show the comparison of up-(↑) and down (↓)-regulated transcripts/proteins in lactate and pyruvate at 6h.

**Figure S3**

**
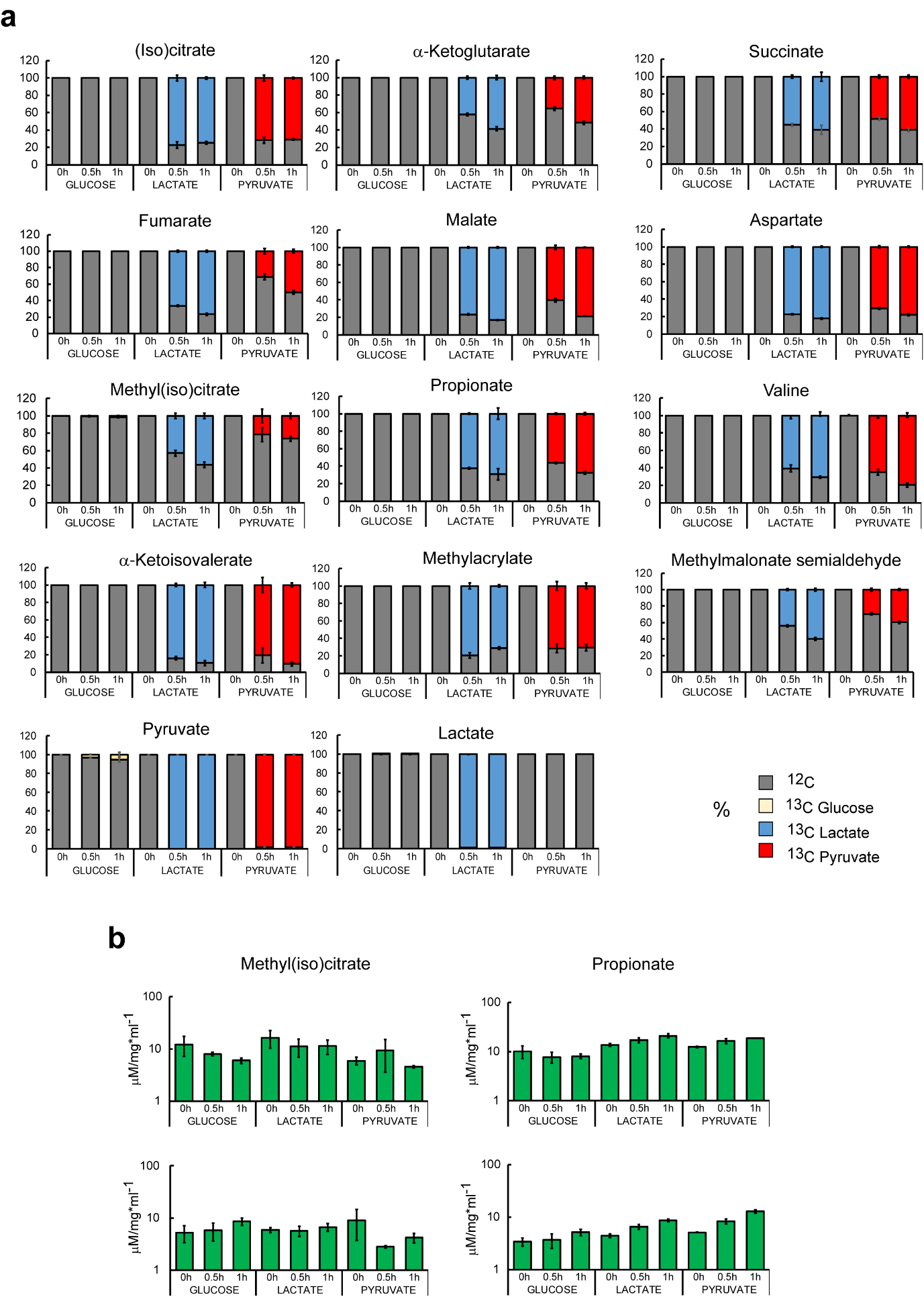
**

**Figure S3. Metabolite total labelling and pool size after 0.5 h and 1 h of exposure to U-13C lactate, pyruvate and glucose.** **a**) Total percentage of ^12^C and ^13^C labelled metabolite from one independent experiment and four biological replicates representative of two independent experiments. The bar charts report the average and standard deviation of the four biological replicates. **b**) Total pool size of methylcitrate and propionate from two independent experiments (experiment 1 at the top; experiment 2 at the bottom) and four biological replicates. The bar charts report the average and standard deviation of the four biological replicates.

**
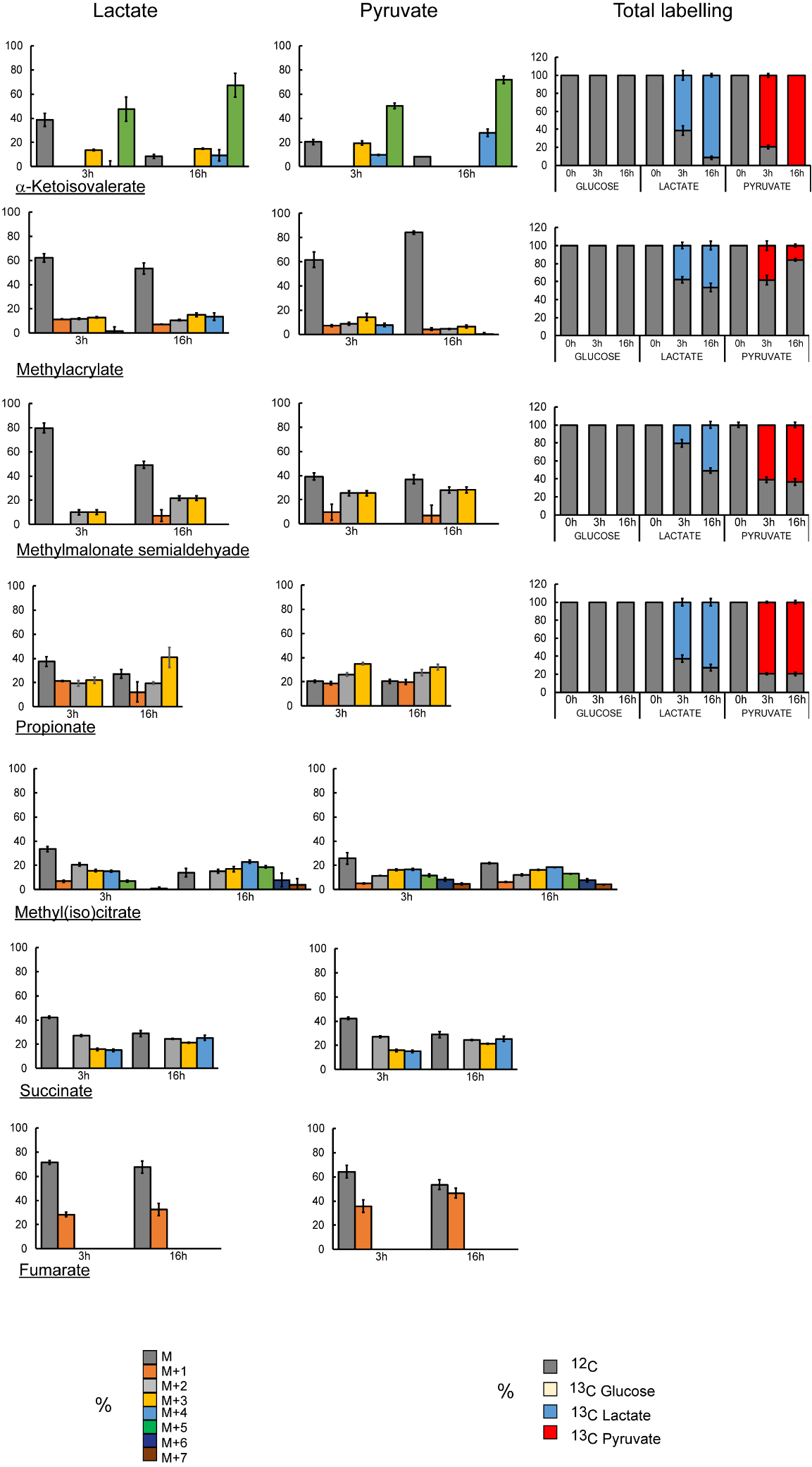
Figure S4**

**Figure S4. Labelling profiles and total labelling of valine metabolism metabolites and intermediates from central carbon metabolism after 3 h and 16 h of exposure to U-13C lactate, pyruvate and glucose.** On the left, percentage of isotopic species of metabolites from valine and central carbon metabolism in lactate and pyruvate. On the right, total percentage of ^12^C and ^13^C in labelled metabolites from valine metabolism. The charts show average and standard deviation of one independent experiment and four biological replicates representative of three independent experiments.

**Figure S5**

**
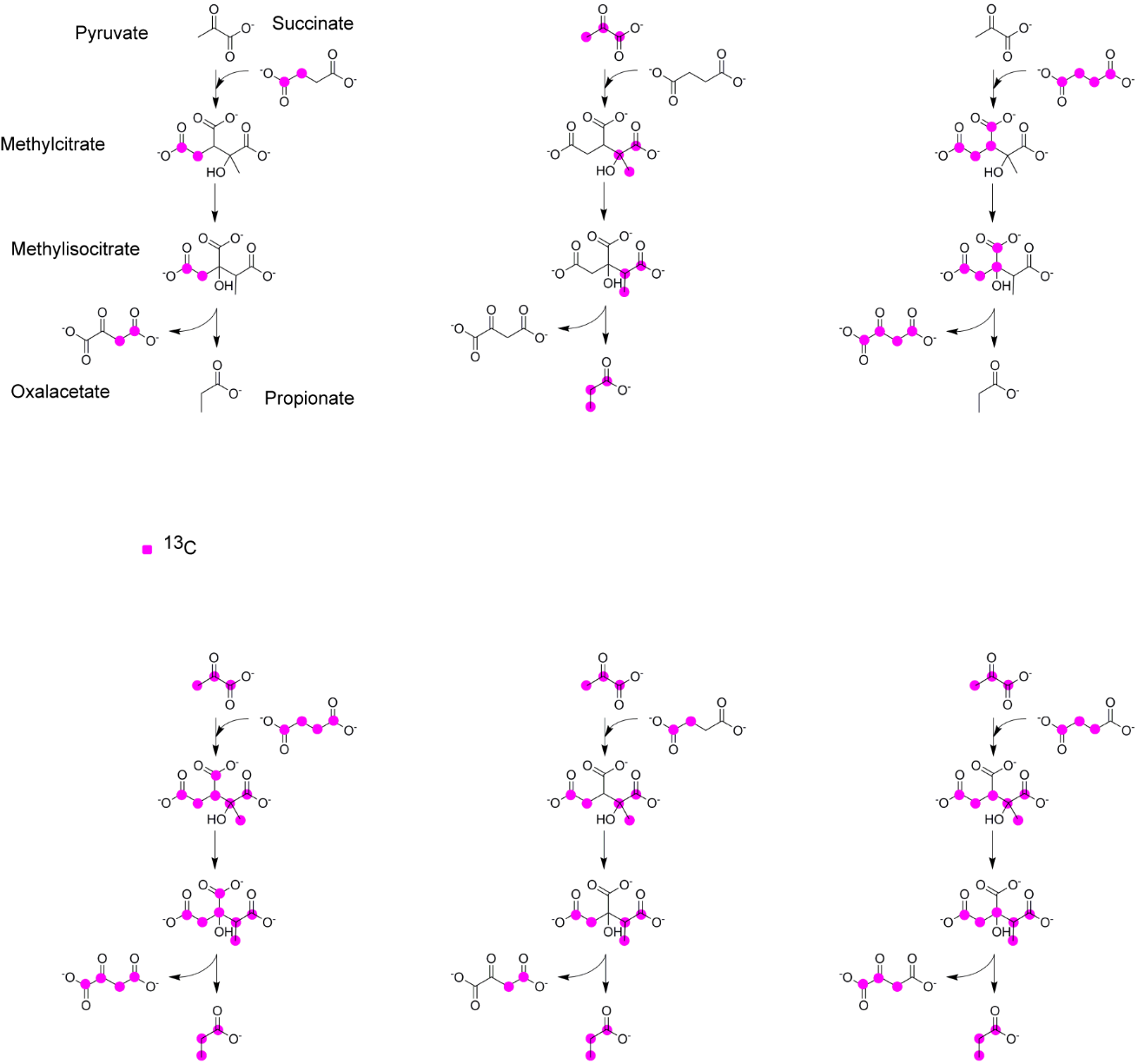
**

**Figure S5. ^13^C incorporation from pyruvate to methyl-citrate.** The six panels illustrate the reactions of the methylcitrate cycle involving methyl(iso)citrate. The metabolite names are indicated only in the first panel. Each panel displays the incorporation of ^13^C across the pathway from pyruvate and succinate to propionate and oxalacetate. Only the reactions producing the most abundant methyl(iso)citrate isotopic species found in our metabolomics experiments are reported.

**Figure S6
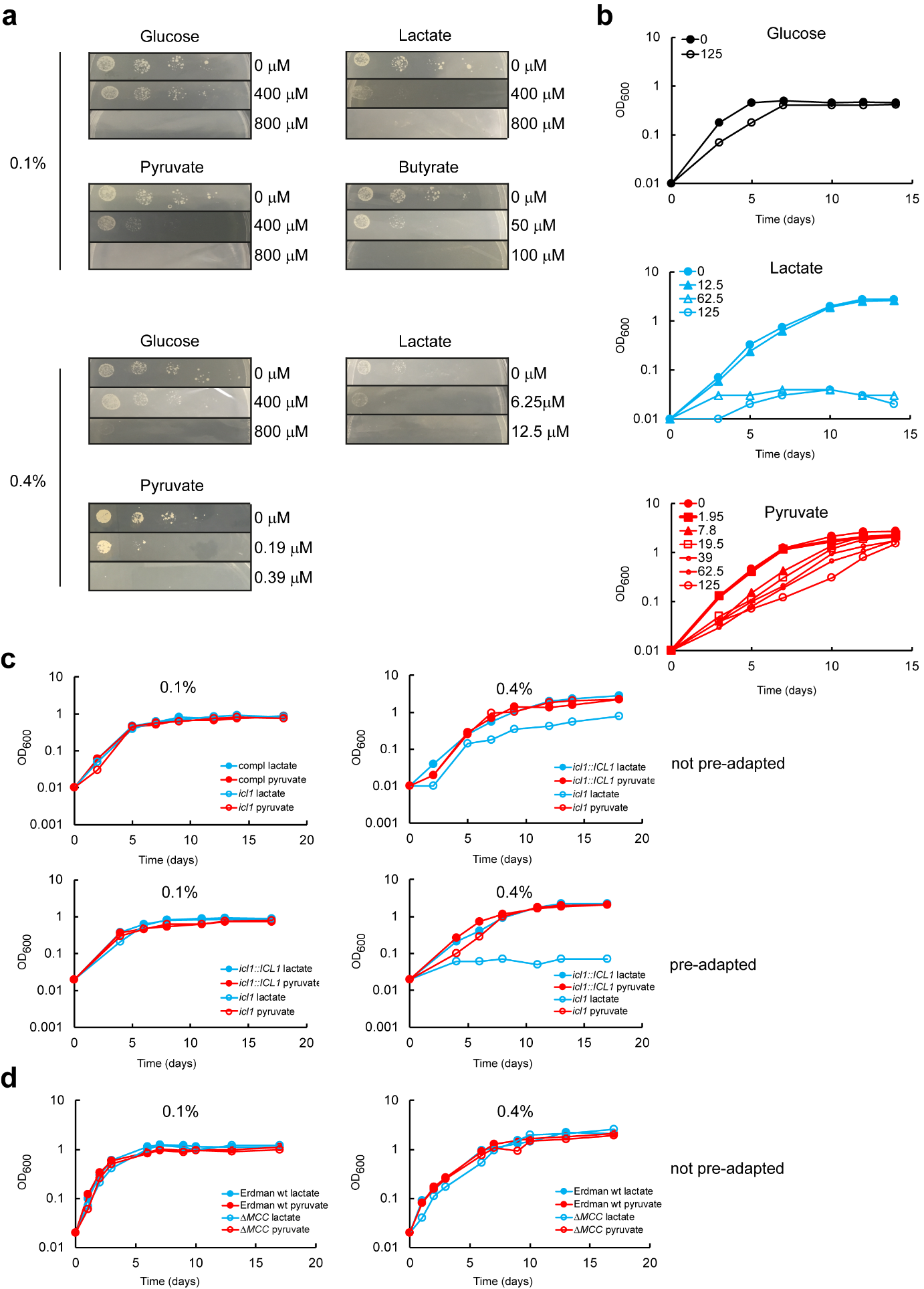
**

**Figure S6.** **H37Rv** **growth inhibition by 3-nitropropionate in lactate, pyruvate and glucose. a)** 5µl of serial dilutions of 0.1 % carbon source pre-adapted cultures were spotted on solid media supplemented with the specific sole carbon source and different concentration of 3NP. Pictures were taken after 30 days of growth, and they represent one of 2 independent experiments. **b)** Growth in 0.4 % carbon source and different concentration of 3NP. The plots show the results of one experiment representative of 2-3 independents. The cultures were pre-adapted in 0.2 % carbon source. The concentration of 3NP is in µM. **c**) Growth of an H37Rv *icl1* mutant and its complemented strain in 0.1 and 0.4 % lactate and pyruvate. The top charts show the growth of not pre-adapted cultures; the charts at the bottom show the growth after pre-adaptation at 0.1% carbon source. The results are representative of one experiment independent.**d)** Growth of a not pre-adapted *prpDC* mutant (Erdman background) and its parental in 0.1 and 0.4 % lactate and pyruvate. The results are representative of one independent experiment.

**Figure S7**

**
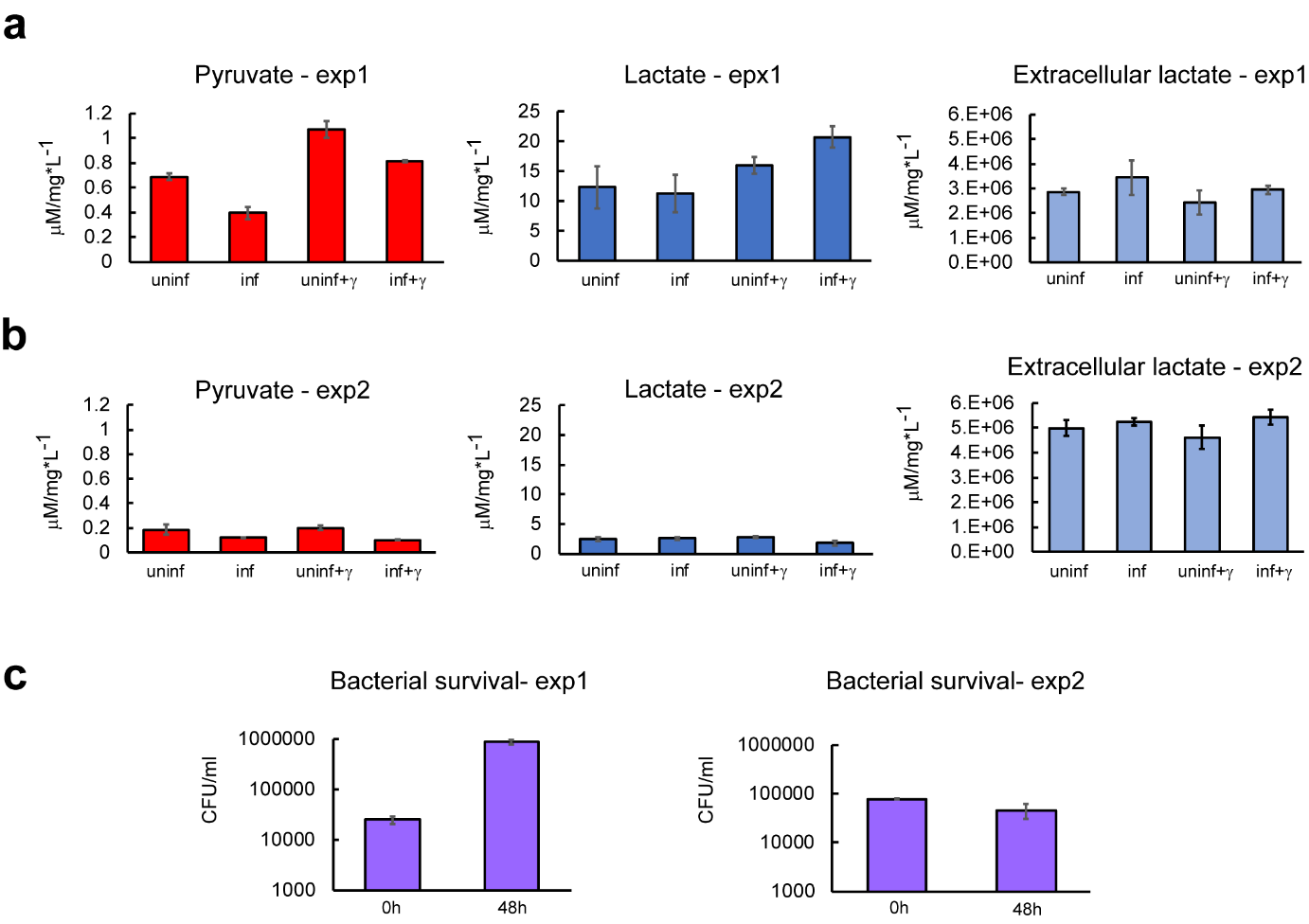
**

**Figure S7. Detection of lactate and pyruvate content in blood-derived human macrophages infected with H37RV. a)** Pyruvate and lactate concentration in 3*10^6^ macrophages. **b**) Secreted lactate concentration from 10^6^ macrophages. The concentration is reported as molarity normalized with residual protein concentration. **c**) Mtb bacterial survival in resting macrophage infection is showed (similar survival in INFγ activated macrophages).

**
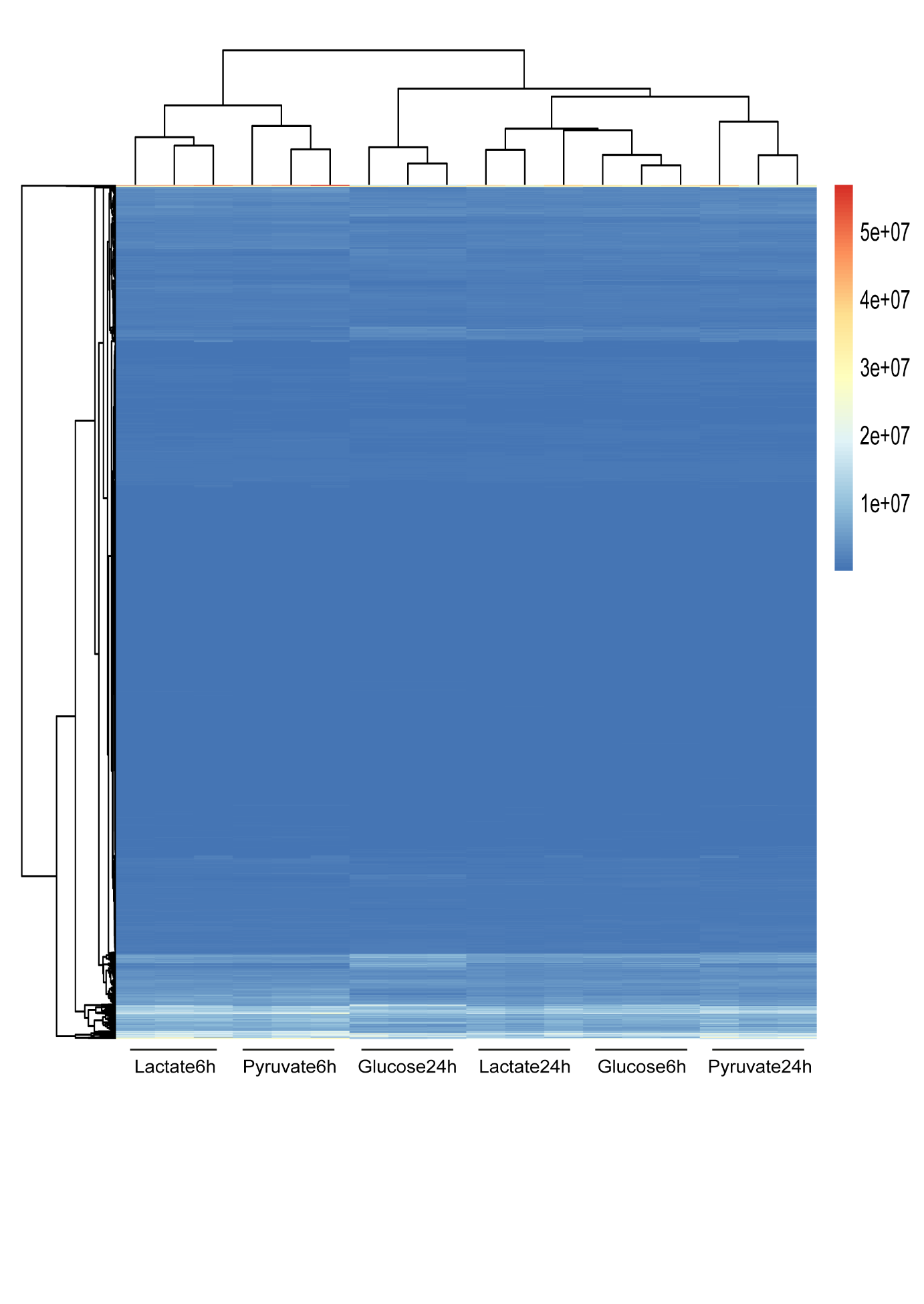
Figure S8**

**Figure S8. Transcriptomics data reproducibility.** The heatmap was obtained clustering the raw reads from three independent experiments by gene (vertical) and sample (horizontal). The outliers identified in figure S9 are excluded.

**
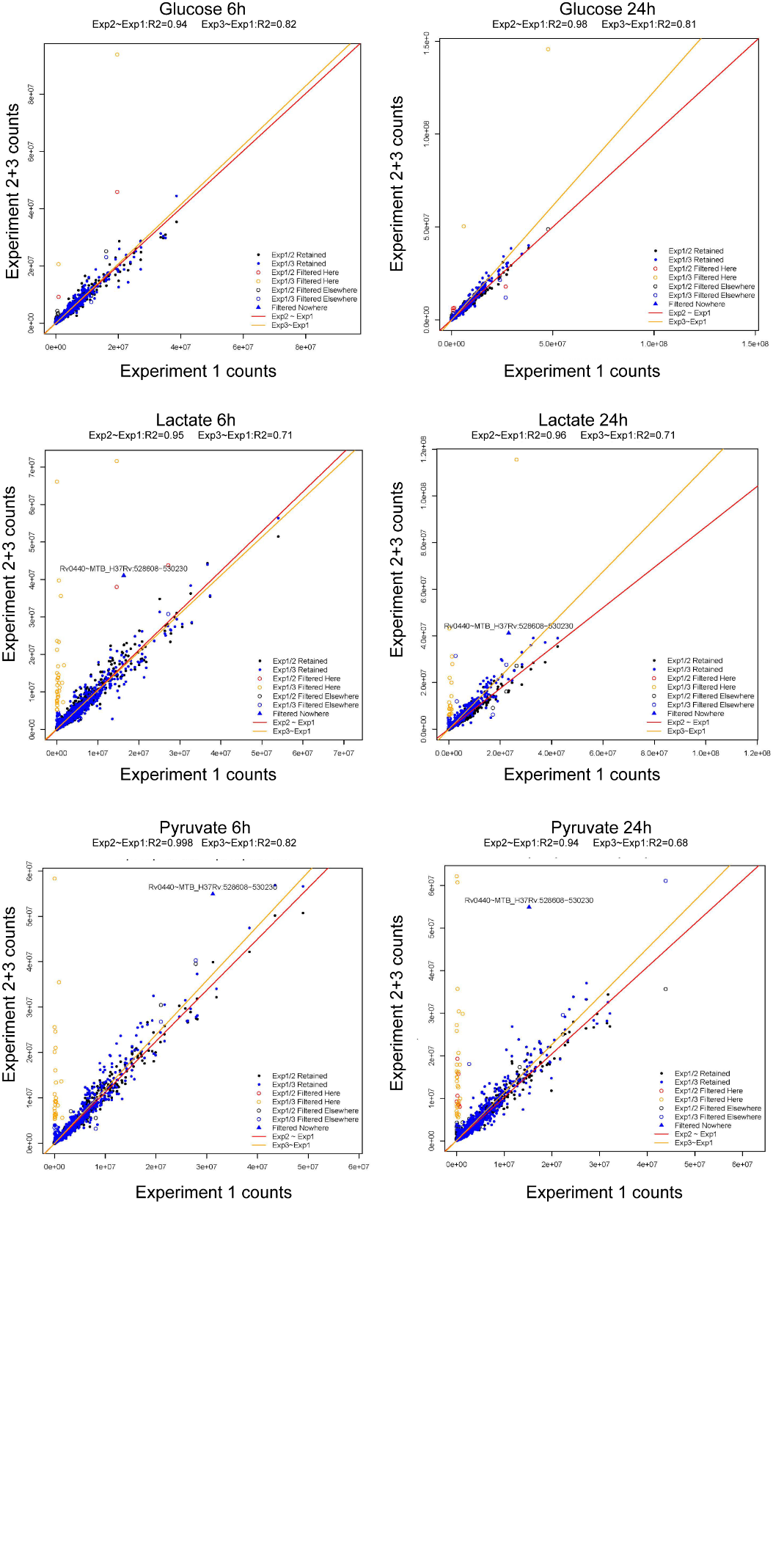
Figure S9**

**Figure S9. Pair-wise regression of within condition replicates for transcriptomics data**. Per each transcript, the reads from experiment 1 were plotted against reads from experiment 2 and 3. The yellow and light brown dots represent the outliers genes excluded from the final analysis.

**Figure S10**

**
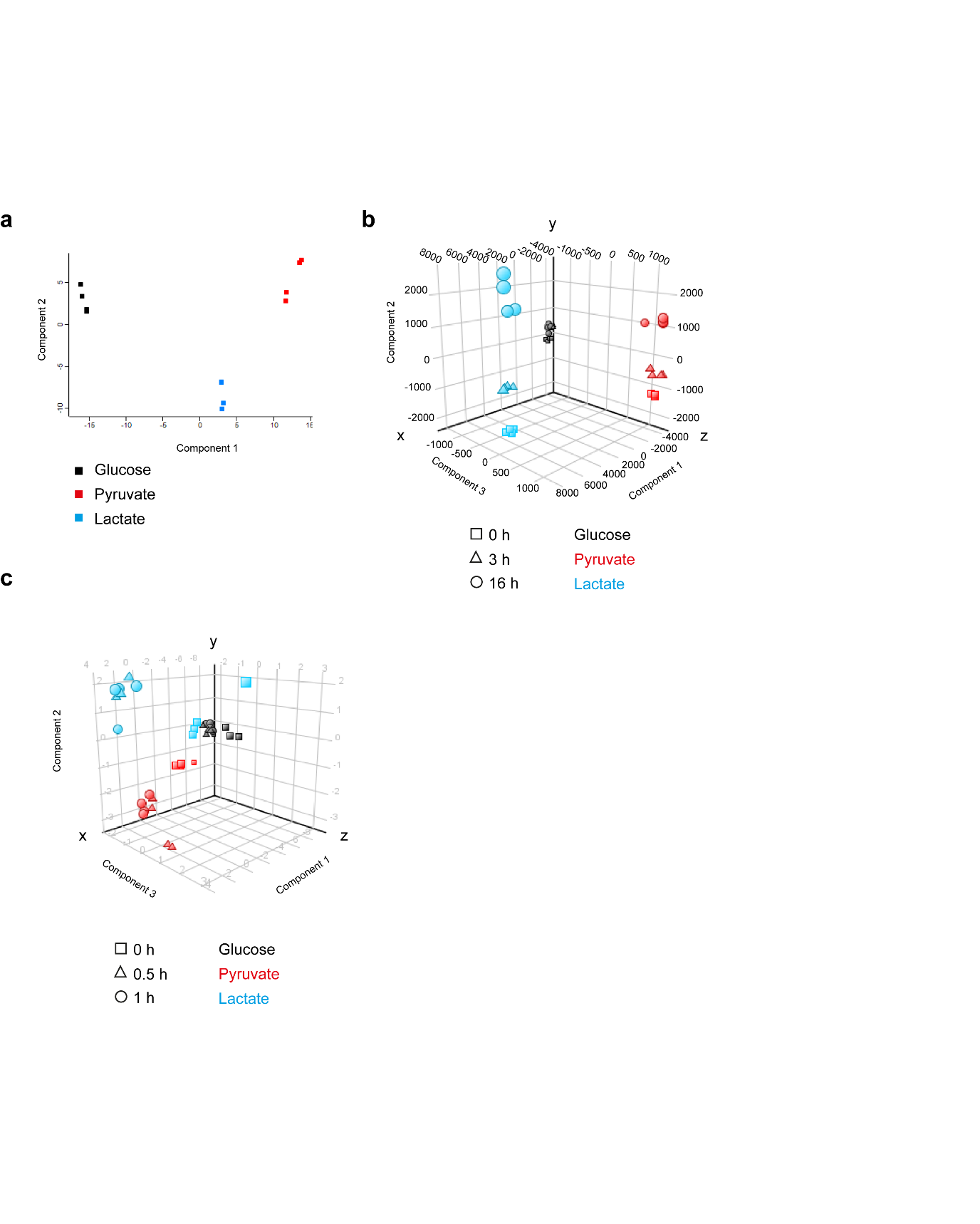
**

**Figure S10. Proteomics and metabolomics principal component analysis (pca). a)** Proteomics data pca from two independent experiments and two LC-MS technical replicates. **b**) 3 h and 16 h metabolomics data pca from one independent experiment and four replicates for each condition. It is representative of three independent experiments. **c**) 0.5 h and 1 h metabolomics data pca from one independent experiment and four replicates for each condition. It is representative of two independent experiments.

**Supplementary file 1.** List of transcripts up- and down-regulated in lactate and pyruvate vs. glucose.

**Supplementary file 2.** List of proteins significantly up- and down-regulated in lactate and pyruvate vs. glucose.

**Supplementary file 3.** Overlaps of transcripts and proteins between lactate and pyruvate.

**Supplementary file 4.** List of transposon mutants with growth defect in lactate and pyruvate.

**Supplementary file 5.** Summary of lipid metabolism genes with altered expression.

**References for figure 1**

* Ganapathy, U. et al. Two enzymes with redundant fructose bisphosphatase activity sustain gluconeogenesis and virulence in Mycobacterium tuberculosis. Nat Commun 6, 7912 (2015).

** Baughn, A.D., Garforth, S.J., Vilcheze, C. & Jacobs, W.R., Jr. An anaerobic-type alpha-ketoglutarate ferredoxin oxidoreductase completes the oxidative tricarboxylic acid cycle of Mycobacterium tuberculosis. PLoS Pathog 5, e1000662 (2009).

***Maksymiuk, C., Balakrishnan, A., Bryk, R., Rhee, K.Y. & Nathan, C.F. E1 of alpha-ketoglutarate dehydrogenase defends Mycobacterium tuberculosis against glutamate anaplerosis and nitroxidative stress. Proc Natl Acad Sci U S A 112, E5834-5843 (2015).

**Supplementary Methods**

***Growth in different aeration condition***

H37Rv strain was pre-adapted in 10 mL of 7H9 supplemented with 0.1 % or 0.2 % of each carbon source in 50 mL Falcon tube at 30 rpm. Cultures at mid-log phase were diluted to an OD of 0.025 in 7H9 in different aeration conditions: a) 15 mL in 50 mL Falcon tube at 30 rpm; b) 15 ml in 150ml square bottle without shaking. c)17 mL in 25 mL screw-capped bacteriological glass tubes stirring with magnets at 130 rpm ^1^; each experiment was performed in quadruplicate, one of them was used as oxygen-consuming control adding methylene blue^1^. The growth was monitored measuring the absorbance at 600 nm. Two independent experiments were performed for each condition.

***Preparation of monocyte-derived macrophages***

Monocytes were isolated from leukocyte cones (product number NC24) provided by National Health Service Blood and Transplant (NHSBT) facility at Colindale Hospital from healthy anonymous donors as previously described^2^. Blood was centrifuged over Ficoll-Paque Premium (GE Healthcare 28-4039-56 AD) to remove red blood cells. White blood cells were then collected and washed to remove platelets. Cell pellets were then resuspended in 10 ml Red Blood Cell Lysing Buffer (Sigma-Aldrich 11814389001) for 20 min to remove residual red blood cells. Cells were then re-suspended in MACS rinsing buffer (Miltenyi 130-091-222) with 0.5% bovine serum albumin to a total volume of 80 μl per 10^7^ cells and incubated with 20 μl per 10^7^ cells of anti-CD14 magnetic beads (Miltenyi 130-050-201) on ice for 20 min. Cells were then washed and magnetically separated on a column (Miltenyi 130-042-401) in a MACS magnetic separator. CD14-positive cells were then pelleted and resuspended in warm RPMI 1640 with GlutaMAX and HEPES (Gibco 72400021) with 9.1% heat-inactivated fetal calf serum (hereafter referred to as ‘complete medium’) and 10 ng/mL granulocyte macrophage-colony stimulating factor (GM-CSF, Miltenyi 130-093-862). Cells were then plated into non-cell culture treated Sterilin petri-dishes at 37°C and 5% CO_2_. Fresh media was added after 3 days. After 3 days, the differentiated macrophages were detached in MACS rinsing buffer and resuspended in warmed complete media with 10 ng/ml GM-CSF and with or without 100 U/ml interferon-gamma (Gibco PHC 4031). 10^6^ cells were plated into each well of a 6-well tissue-culture treated plate which was placed into an incubator overnight. For CFU analysis 2 x 10^5^ cells were plated per well of a 24-well plate, and for macrophage viability analysis 2 x 10^6^ cells were plated per well of a 24-well plate on top of sterile glass coverslips.

***Macrophage infection and cell viability***

Infection was performed as previously described ^2^. Mtb growing at log phase was were pelleted and washed twice with PBS. The pellet was then disrupted by vigorously shaking with 10-15 2 mm glass beads by hand for 1 min to break up clumps. Bacteria were then suspended in warm complete medium and centrifuged for 5 min at 300 g to remove residual clumps. Macrophages were infected at a multiplicity of infection of 5 for 2 h. Macrophage viability was measured as previously described ^2^. Briefly, macrophages were washed once with DPBS and then incubated with 25 μg/ml propidium iodide in complete media for 20 min at room temperature. Cells were then washed with PBS and fixed in 4% paraformaldehyde in PBS overnight. Viability was analysed by confocal fluorescence microscopy. Red fluorescence in the nucleus indicates host cell death. No significant macrophage death was observed. To analyse bacterial survival, macrophages were washed 3 x in PBS then lysed in 100 μl water with 0.05% TWEEN-80. Serial dilutions were plated onto 7H11 agar plates.

**Reference**

Lerner, T.R., Borel, S., Greenwood, D.J., Repnik, U., Russell, M.R., Herbst, S., Jones, M.L., Collinson, L.M., Griffiths, G., and Gutierrez, M.G. (2017) Mycobacterium tuberculosis replicates within necrotic human macrophages. *J Cell Biol* **216**: 583-594.

Wayne, L.G. (2001) In Vitro Model of Hypoxically Induced Nonreplicating Persistence of Mycobacterium tuberculosis. *Methods Mol Med* **54**: 247-269.
